# Fluorescence imaging sheds light on the immune evasion mechanisms of hepatic stellate cells mediated by superoxide anion

**DOI:** 10.1038/s42003-024-06245-y

**Published:** 2024-05-10

**Authors:** Yuantao Mao, Chuanchen Wu, Xin Wang, Fanghui Zhang, Xinru Qi, Xia Li, Ping Li, Bo Tang

**Affiliations:** 1 College of Chemistry, Chemical Engineering and Materials Science, Collaborative Innovation Center of Functionalized Probes for Chemical Imaging in Universities of Shandong, Key Laboratory of Molecular and Nano Probes, Ministry of Education, Institute of Biomedical Sciences, Shandong Normal University, Jinan, 250014 China; 2 Innovative Institute of Chinese Medicine and Pharmacy, Shandong University of Traditional Chinese Medicine, Jinan, China; 3 Laoshan Laboratory, 168 Wenhai Middle Rd, Aoshanwei Jimo, Qingdao, 266237 Shandong China

**Keywords:** Chemical tools, Immune evasion, Cancer microenvironment

## Abstract

Whether and how the reactive oxygen species generated by hepatic stellate cells (HSCs) promote immune evasion of hepatocellular carcinoma (HCC) remains mysterious. Therefore, investigating the function of superoxide anion (O_2_^•−^), the firstly generated reactive oxygen species, during the immune evasion become necessary. In this work, we establish a novel in situ imaging method for visualization of O_2_^•−^ changes in HSCs based on a new two-photon fluorescence probe TPH. TPH comprises recognition group for O_2_^•−^ and HSCs targeting peptides. We observe that O_2_^•−^ in HSCs gradually rose, impairing the infiltration of CD8^+^ T cells in HCC mice. Further studies reveal that the cyclin-dependent kinase 4 is deactivated by O_2_^•−^, and then cause the up-regulation of PD-L1. Our work provides molecular insights into HSC-mediated immune evasion of HCC, which may represent potential targets for HCC immunotherapy.

## Abstract (web summary)

Using two-photon fluorescence imaging, the authors show that superoxide anion in hepatic stellate cells impairs the infiltration of CD8+ T cells in a mouse model of hepatocellular carcinoma.

## Introduction

Hepatocellular carcinoma (HCC) is the fourth leading cause of cancer-related death, posing a serious threat to people’s health and lives^1,2^. In recent decades, a variety of immunotherapy strategies have been developed for the treatment of HCC^3,4^. Although have shown great promises, their clinical therapeutic efficacy was reduced by the immune evasion^3^. Immune evasion refers to the process through which cancer cells inhibit or decrease the body’s immune response to malignancy. Undoubtedly, immune evasion is regarded as an indispensable strategy for HCC cell survival^5,6^. Immune evasion in HCC complicates the treatment, and also increases the risk of tumor metastasis and postoperative recurrence. Therefore, understanding the molecular mechanism of immune evasion can provide vital insight for HCC treatment.

Hepatic stellate cells (HSCs) account for 10% of the nonparenchymal cells in the liver. HSCs are crucial mediators in regulating the physiological and pathological processes of liver^7^. When stimulated by injuries or immune responses, the HSCs in quiescent state transform into active state (aHSCs)^8^. Subsequently, aHSCs regulate liver fibrosis and immune processes via secreting active molecules such as the extracellular matri, cytokines, and reactive oxygen species (ROS) through lipid metabolism^9,10^. Current studies have shown that abnormal aHSCs could cause the immune evasion of HCC cells^11–13^. Coulouarn et al. found that HCC cells upregulated the expression of vascular endothelial growth factor A in HSCs, causing the promotion of angiogenesis at tumor sites^12^. In addition, aHSCs promoted migration and invasion of HCC cells through focal adhesion kinase-matrix metalloproteinase 9 signaling^13^. However, the detailed molecular mechanisms still need to be explored.

Superoxide anion (O_2_^•−^), as the first ROS produced in organisms, profoundly affects the immune responses of HCC^14,15^. The activation of HSCs is accompanied by lipid metabolism and oxidative phosphorylation, providing energy to maintain the normal physiological functions of the cells. Importantly, oxidative phosphorylation is an important pathway to produce O_2_^•−^. Therefore, we assume that during this process, the levels of O_2_^•−^ may change distinctly. However, due to the high activity and short duration of O_2_^•−^, no suitable method for real-time and in situ detecting O_2_^•−^ in HSCs has been reported. Thus, whether and how O_2_^•−^ produced in aHSCs regulate immune evasion of HCC remains mysterious. Therefore, it is urgent to develop a tool that can be used to explore the flux of O_2_^•−^ in HSCs, and investigate the detailed mechanism of HCC immune evasion mediated by O_2_^•−^.

Currently, two-photon fluorescence imaging technique with high sensitivity and real-time detection has served as a superior approach to monitor molecule events in vitro and in vivo^16–18^. This technique has excellent repeatability, and can greatly avoid the interference of biological tissue absorption by its two-photon absorption property. With the help of two-photon fluorescence imaging technique, our group had observed the dynamic, reversible changes of O_2_^•−^ in the liver injury mediated by ischemia-reperfusion and in brains of mice with depression^17,18^. However, there is still a lack of imaging material for two-photon fluorescence imaging of O_2_^•−^ level changes in HSCs.

**Fig. 1:**
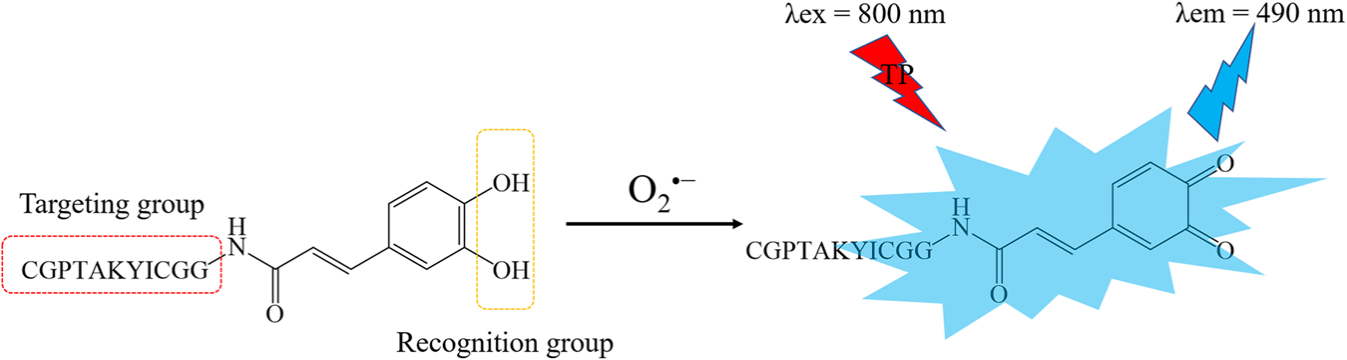
Structure and recognition mechanism of TPH. The catechol unit of the caffeic acid in the probe is an electron-donating group, and upon reaction with O_2_^•−^, the catechol is oxidized to an electron-absorbing quinone structure, which redistributes the electrons within the molecule, resulting in intense fluorescence.

To solve the above problems, we designed a two-photon fluorescence probe TPH for imaging O_2_^•−^ in HSCs. TPH was composed of CGPTAKYIC^19^ for HSCs-specific targeting and caffeic acid residue as a recognition group for specific detection of O_2_^•−^. In the presence of O_2_^•−^, caffeic acid residues undergo phenol–quinone tautomerization, resulting in enhanced fluorescence (Fig. 1). Using TPH, we investigated the function of O_2_^•−^ during the activation of HSCs in vitro and in vivo. Further research found that excess O_2_^•−^ caused dysfunction of cyclin-dependent kinase 4 (CDK4), which in turn led to increased programmed cell death-ligand 1 (PD-L1) levels. Ultimately, these changes promoted the immune evasion of HCC.

## Results and discussion

### Optical properties of TPH

**Fig. 2:**
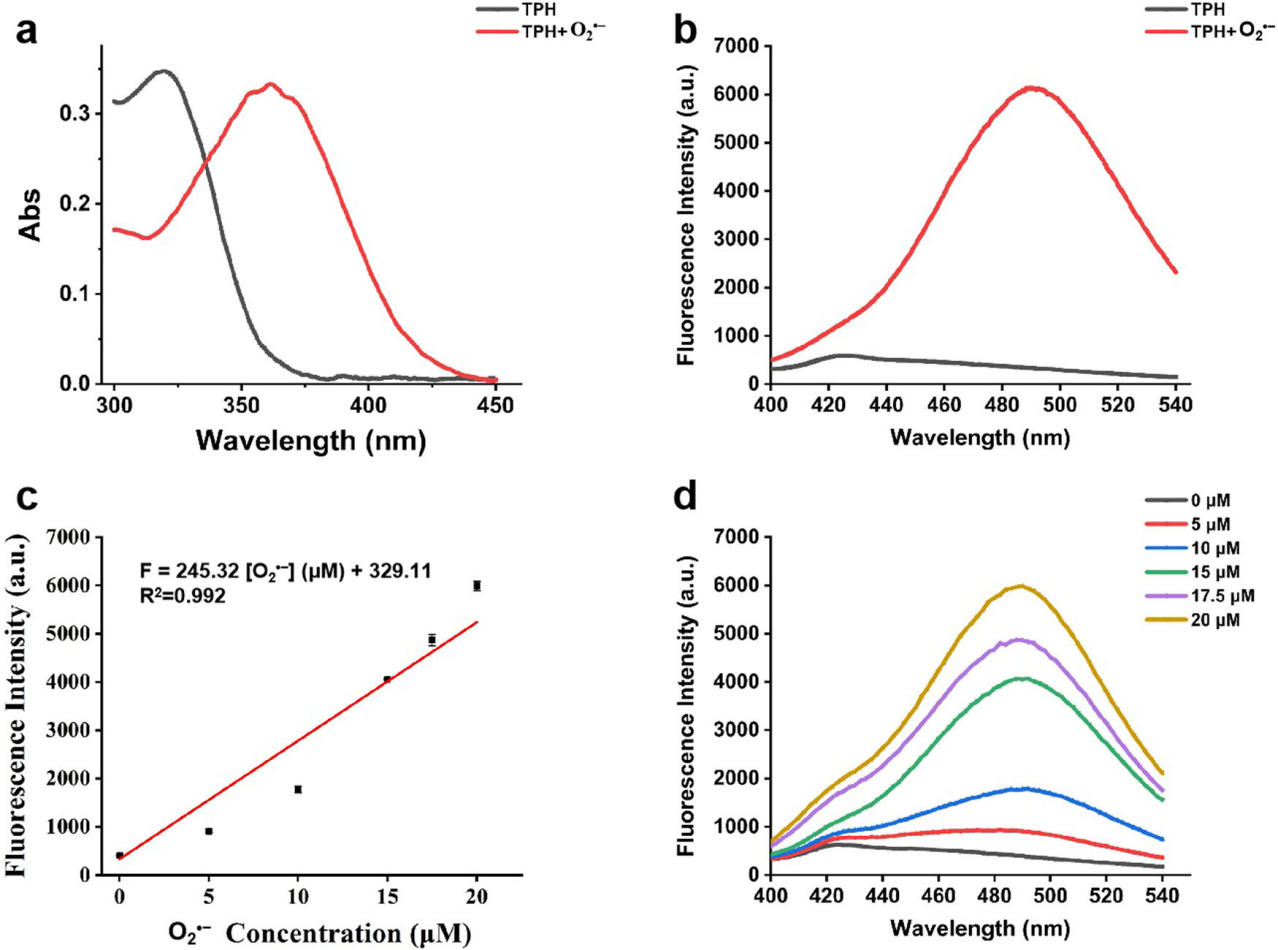
Optical properties of TPH. **a**, **b** Absorption and fluorescence spectra before and after the reaction of 20 μM TPH with 20 μM O_2_^•−^. **c** Linear relationship between fluorescence intensities and O_2_^•−^ concentrations (0–20 μM). **d** Fluorescence spectra of 20 μM TPH in response to various concentrations of O_2_^•−^.

The synthesis and structural characterizations of TPH were shown in Supplementary Figs. 1–3. Prior to the in vivo application, we investigated the photophysical properties of TPH. As shown in Fig. 2a, the absorption maximum peak of TPH was 320 nm, which would shift to 370 nm after reacting with O_2_^•−^. Next, the fluorescence response of TPH to O_2_^•−^ was tested. The addition of O_2_^•−^ resulted in an increased fluorescence intensity due to the oxidation of the phenolic hydroxyl group in TPH by O_2_^•−^ (Fig. 2b). The fluorescence intensities of TPH at 490 nm increased gradually as O_2_^•−^ was added in the range of 0–20 μM. The linear equation was *F* = 245.32 [O_2_^•−^] (μM) + 329.11, with a linear correlation coefficient of 0.992. The limit of detection (LOD) was 111 nM, as calculated by the equation LOD = 3*σ*/*K* (Fig. 2c, d). To interrogate the specificity of TPH toward O_2_^•−^, we examined the fluorescence responses of TPH to O_2_^•−^ and other common interfering substances under simulated physiological conditions. The probe can also recognize O_2_^•−^ under two-photon excitation of 800 nm (Supplementary Fig. 4). Encouragingly, only O_2_^•−^ resulted in a significant increase of the fluorescence signal at 490 nm without any interference from other highly active molecules in vivo (Supplementary Fig. 5). This suggested that TPH showed excellent specificity to O_2_^•−^. Moreover, TPH exhibits the reversible response to O_2_^•−^. In addition, TPH possessed other advantages including instantaneous response, good photostability and favorable biocompatibility (Supplementary Figs. 6–12). The above results highlight that the probe could be a promising candidate for O_2_^•−^ detection during the activation of HSCs.

### O_2_^•−^ detection during the activation of HSCs

**Fig. 3:**
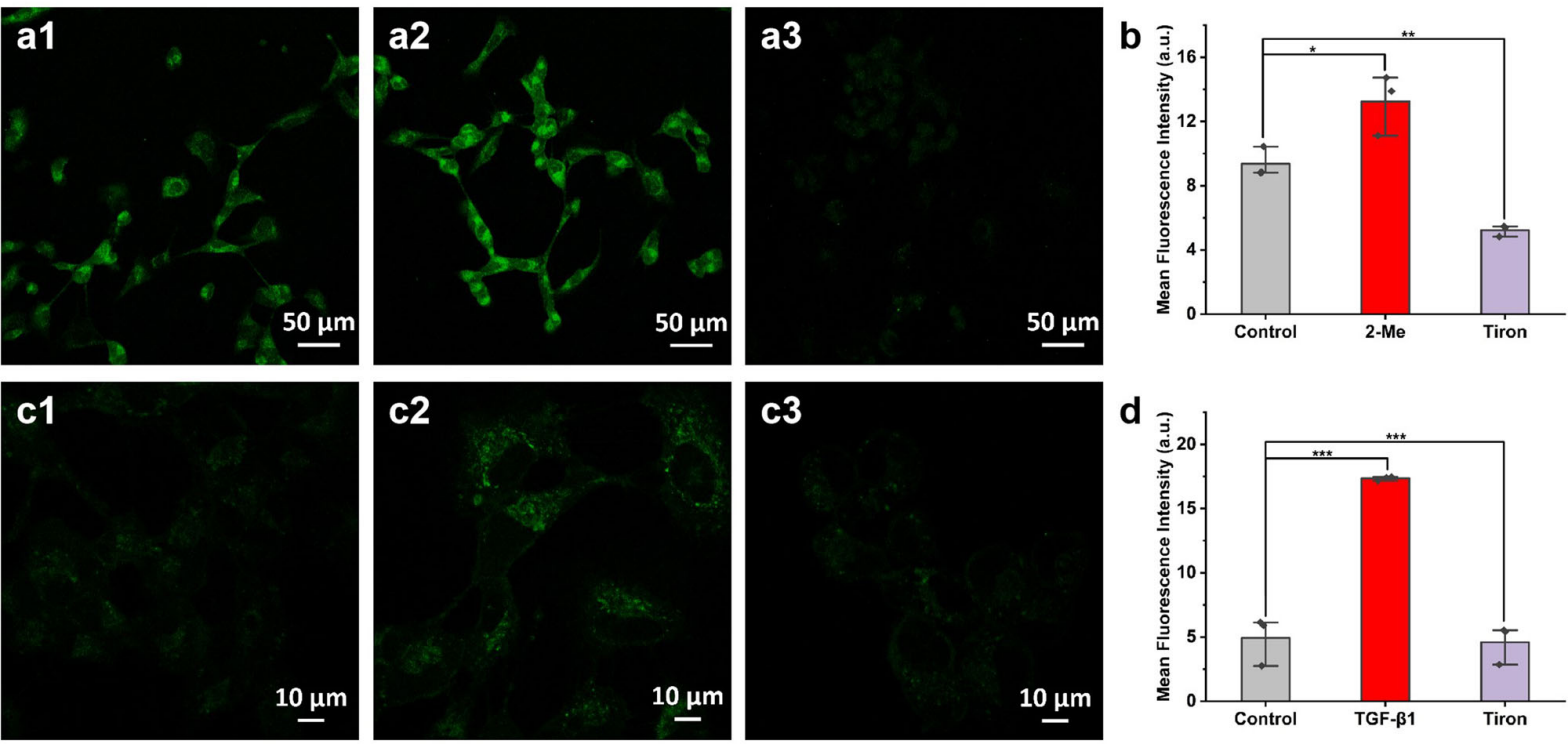
O_2_^•−^ fluorescence imaging of LX-2 cells. **a** O_2_^•−^ fluorescence imaging of endogenous O_2_^•−^ in LX-2 cells. a1: control; a2: 2-Me (0.1 µg mL^−1^) for 15 min; a3: after 2-Me incubation, Tiron (10 µM) was added and incubated for 30 min. **c** O_2_^•−^ fluorescence imaging of LX-2 cell activation. c1: control; c2: TGF-β1 (5 ng mL^−1^) was incubated for 12 h; c3: TGF-β1 was incubated and Tiron (10 µM) was added for 30 min. **b**, **d** Fluorescence intensities of (**a**) and (**c**). Scale bar = 50 μm (**a**) or 10 μm (**c**). The data were expressed as mean ± SD, *n* = 3. *p < 0.01, **p < 0.05, ***p < 0.001 compared to the control group.

Motivated by the preeminent photophysical properties of TPH, we explored its capability of imaging O_2_^•−^ during the activation of HSCs. Cell selectivity experiments confirmed that TPH could specifically target HSCs (Supplementary Fig. 13). First, we verified whether the TPH could recognize O_2_^•−^ in HSCs. LX-2 cells were pretreated with 2-methoxyestradiol (2-Me) to boost the endogenous O_2_^•−^ level^20,21^. As shown in Fig. 3a, b, compared with the control group, 2-Me treated cells elicited brighter fluorescence. In contrast, the fluorescence intensity was significantly reduced when Tiron (O_2_^•−^ scavenger) was added to those cells^22^. These images illustrated that O_2_^•−^ in HSCs could be tested by TPH. Subsequently, we explored the changes of O_2_^•−^ levels during the activation of HSCs. We successfully activated hepatic stellate cells using TGF-β1 (Supplementary Fig. 14)^23^. As displayed in Fig. 3c, d, cells with TGF-β1 incubation showed significantly increased fluorescence compared with the control group. Moreover, the fluorescence intensity of the Tiron group was lower than that of the TGF-β1 group. Moreover, the imaging results of O_2_^•−^ level changes during HSCs activation could be stimulated performed under both one-photon and two-photon excitations (Supplementary Fig. 15).

The finding indicated that a large amount of O_2_^•−^ was produced during the activation of HSCs. Overall, we successfully established an imaging method for detecting O_2_^•−^ within HSCs.

### The activation degrees of HSCs and the levels of intracellular O_2_^•−^

**Fig. 4:**
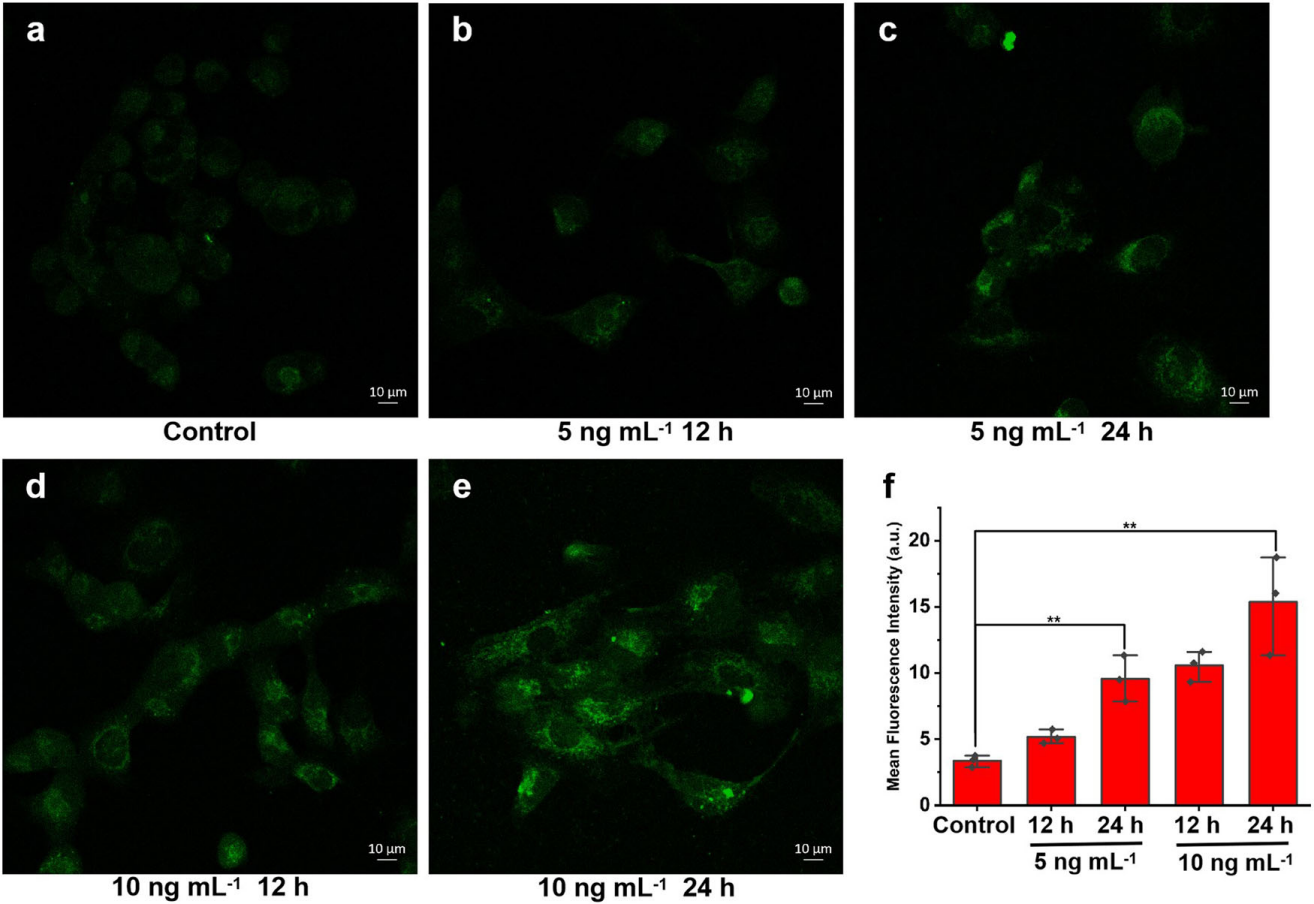
O_2_^•−^ fluorescence imaging of LX-2 cells with different degrees of activation. **a**–**e** The LX-2 cells were incubated with different TGF-β1 concentrations or times. **f** Fluorescence intensities of **a**–**e**. Scale bar = 10 μm.The data were expressed as mean ± SD, *n* = 3. **p < 0.05 compared to the control group.

Next, the relationship between the activation degrees of HSCs and the O_2_^•−^ levels was explored by the established imaging method. To build the HSCs with varying activation degrees, LX-2 cells were pretreated with different stimulation conditions. As illustrated in Fig. 4, with the increasing of concentration or incubation time, the stronger fluorescence was observed. The above data revealed that the activation degree of HSCs was positively correlated with O_2_^•−^ level.

### O_2_^•−^ imaging in mouse HSCs with different activation degrees

**Fig. 5:**
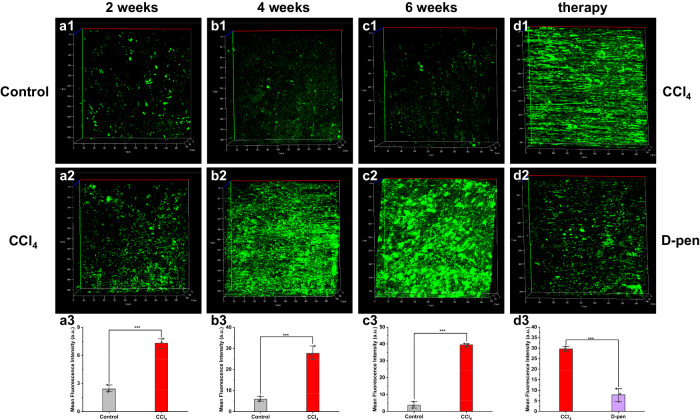
Fluorescence imaging of mice with different levels of HSCs activation. **a**–**c** Mice were intraperitoneally injected with olive oil or CCl_4_ solution for 2, 4, and 6 weeks, respectively. Control: olive oil; CCl_4_: CCl_4_ solution. **d** Fluorescence imaging of mice with inhibition of HSCs activation. The mice were first given CCl_4_ solution for 6 weeks, followed by 2 weeks of either stroke-physiological saline solution or D-pen. The data were expressed as mean ± SD, *n* = 3. ***p < 0.001 compared to the control group.

Encouraged by the results of the cell experiments, we continued to explore the potential of TPH applications in vivo. The mice with various levels of aHSCs were obtained by intraperitoneal injection carbon tetrachloride (CCl_4_) solution (Supplementary Fig. 16)^10,24^. Next, TPH was injected intraperitoneally into the mice before confocal microscope imaging. Compared with the control groups, brighter fluorescence signals were observed in mice with more aHSCs (Fig. 5a–c). These meant that the levels of O_2_^•−^ were increased in mice with aHSCs. Of note, the fluorescence intensities were enhanced 3.0-fold (2 weeks), 4.7-fold (4 weeks), and 10.6-fold (6 weeks), respectively. These findings revealed that as the activation degree of mouse HSCs increased, the O_2_^•−^ levels in HSCs enhanced. Finally, D-penicillamine (D-pen) and tocopherol (VE) were used to inhibit the activation of HSCs^25,26^. A weaker fluorescence was observed in mice with D-pen or VE treated than in mice with CCl_4_ only (Fig. 5d and Supplementary Fig. 17). This result suggested that inhibiting the activation of mouse HSCs could reduce the concentration of O_2_^•−^. Collectively, all these data implied that TPH could be used for the detection of O_2_^•−^ in mouse HSCs. With the help of TPH, we find that aHSCs produced the excessive O_2_^•−^ in mice.

### The O_2_^•−^ level and the immunosuppressive ability of HCC microenvironment

**Fig. 6:**
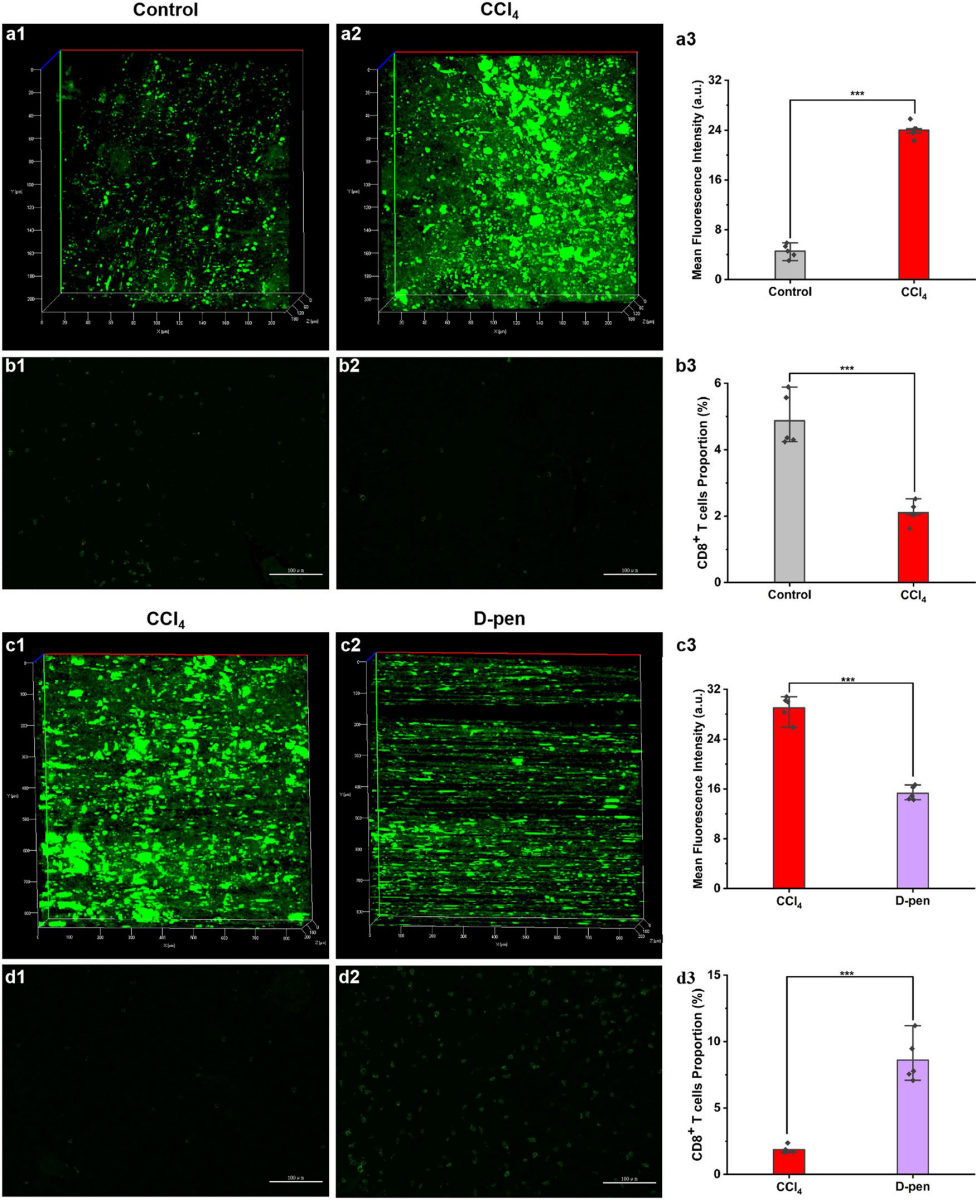
O_2_^•−^ imaging and immunofluorescence staining of HCC mouse models with varying degrees of HSCs activation. **a**, **b** O_2_^•−^ imaging and immunofluorescence staining of ordinary HCC mice and HCC mice with aHSCs. **c**, **d** O_2_^•−^ imaging and immunofluorescence staining of HCC mice with the inhibited aHSCs by D-pen. *λ*_ex_ = 800 nm, *λ*_em_ = 450–550 nm. Scale bar = 100 μm. The data were expressed as mean ± SD, *n* = 5. ***p < 0.001 compared to the control group.

Researches established a closely link between O_2_^•−^ and the immunosuppressive microenvironment of HCC^15,27^. The anti-tumor activity of CD8^+^ T cells inside HCC is affected by ROS^28–30^. Considering the excessive O_2_^•−^ in the aHSCs of mice, we investigated the relationship between O_2_^•−^ in aHSCs and the activity of CD8^+^ T cells in HCC mice. We constructed orthotopic mouse models of HCC containing quiescent-HSCs or aHSCs, and performed fluorescence imaging and immunofluorescence imaging. As shown in Fig. 6a, c, the fluorescence intensities of aHSCs–HCC mice were significantly higher than that of control HCC mice. On the contrary, the proportions of CD8^+^ T cells around the tumor were decreased in aHSCs–HCC mice. This suggests a negative correlation between the two biological events: inhibition of HSCs activation decreased the level of O_2_^•−^ and increased the proportion of CD8^+^ T cells around the tumor (Fig. 6b, d and Supplementary Figs. 18–20). These results attested that the O_2_^•−^ level in HSCs was positively correlated with the immunosuppressive ability of the HCC microenvironment.

It is important that the tumor volume and weight of aHSCs–HCC mice were significantly higher than those of control HCC mice. Meanwhile, using D-pen or VE to reduce the degree of HSCs activation could obviously inhibit the growth of group tumor (Supplementary Fig. 21). This suggests that aHSCs can promote tumor growth. To prove that immune evasion is primarily induced by ROS production, we performed experiments to validate the role of ROS on immune evasion. The experimental results showed that the tumor volume and weight of the mice in the experimental group were smaller compared to the control group (Supplementary Fig. 22). This indicates that scavenging ROS can inhibit tumor growth to a certain extent. All in all, we found that the excess O_2_^•−^ generated by the aHSCs enhanced the immunosuppressive ability of the HCC microenvironment, which in turn affects the growth and development of tumor in mice.

### Mechanism of immune evasion mediated by HSCs O_2_^•−^ in HCC

**Fig. 7:**
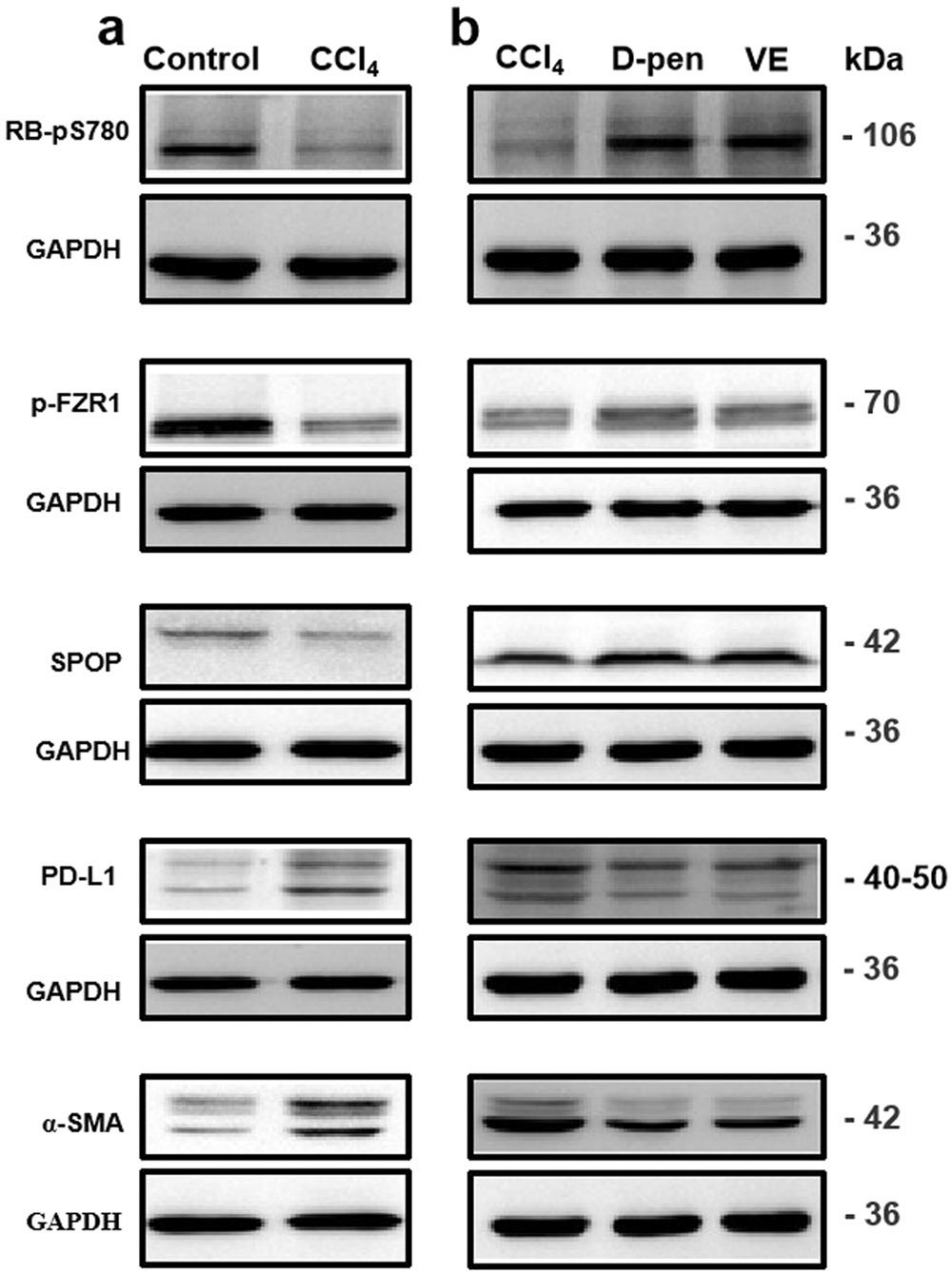
Western blotting of mouse HCC models with different activation levels of HSCs. **a** Ordinary HCC mice and HCC mice with aHSCs. **b** HSCs activation was inhibited in HCC mice.

Inspired by the above-mentioned exciting findings, we explored the molecular mechanism by which O_2_^•−^ regulates the immune evasion of HCC. In view of upregulation of PD-L1 resulting in immune evasion, we first investigated the expression of PD-L1 during the activation of HSCs. As shown in Fig. 7, we found an obvious upregulation of PD-L1 after HSCs were activated in orthotopic mouse models of HCC. Meanwhile, inhibiting the activation of HSCs led to a distinctly downregulation of PD-L1. Collectively, these results demonstrated that PD-L1 was involved in aHSCs-mediated immune evasion of HCC. The PD-L1 blocking antibody treatment experiments results showed that the tumor volume and weight of mice in the PD-L1 group were significantly reduced (Supplementary Fig. 23). This result suggests that the use of PD-L1 inhibitors can significantly inhibit the growth of tumor in mice, revealing the essential role of PD-L1 in immune evasion.

Next, we explored the signaling pathway leading to the expression change of PD-L1 in aHSCs. Previous studies have indicated that CDK4 could regulate the expression of PD-L1 through the CDK4/p-FZR1/SPOP/PD-L1 mechanism^31^. Therefore, we analyzed the effects of changes in HSCs status on CDK4 activity and related molecular levels. Compared with control HCC mice, we noted that CDK4 activity and p-FZR1 levels were decreased after HSCs activation. Low p-FZR1 levels could induce SPOP protein degradation and ultimately promote the upregulation of PD-L1 level (Fig. 7a). In addition, inhibition of HSCs activation could increase CDK4 activity and decrease PD-L1 expression level (Fig. 7b). Similarly, clearance of ROS produced by hepatic stellate cell activation can regulate CDK4 activity and PD-L1 levels. These results elucidated that the mechanism of CDK4 mediating PD-L1 was regulated by the activity of HSCs.

**Fig. 8:**
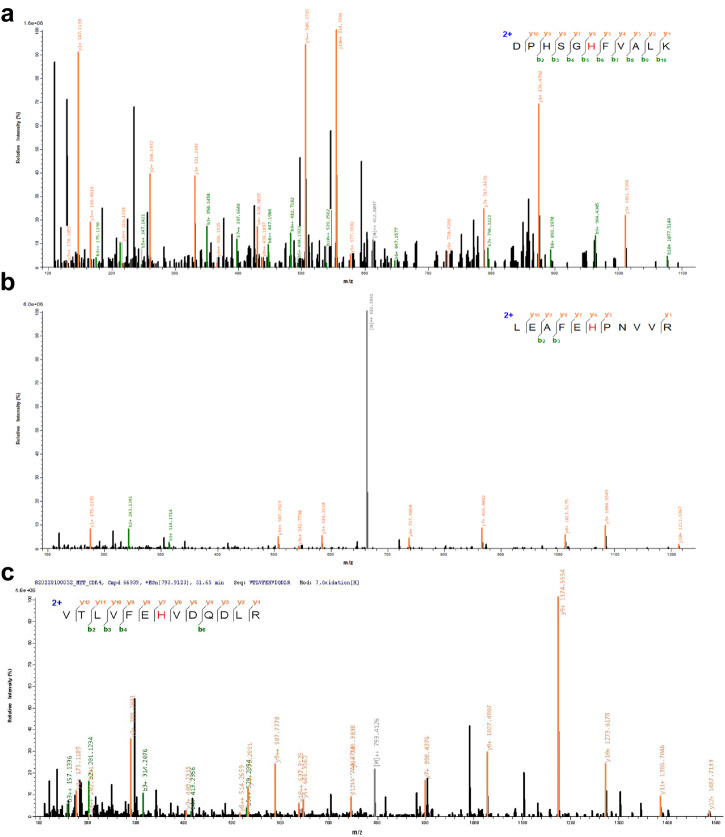
Protein mass spectrometry analysis. **a** His 30. **b** His 68. **c** His 95.

However, what cause the change of CDK4 activity remains unknown. Previous reports suggest that O_2_^•−^ can damage protein activity by oxidizing amino acid residues^17^. Therefore, we hypothesized that O_2_^•−^ in aHSCs might damage CDK4 activity, thereby promoting the immune evasion of HCC. To put our hypothesis to the test, we conducted proteomic analyses through LC–MS/MS to investigate the post-translational modifications of CDK4 by O_2_^•−^. As shown in Fig. 8, three histidine residues (H30, H68, and H95)^32,33^ in the active region of CDK4 were oxidized, which should be responsible for the inactivation of CDK4. Based on the above experimental results, we finally proposed a new detailed mechanism by which HSCs promote the immune evasion of HCC. The excess O_2_^•−^ produced by aHSCs damaged the activity of CDK4, increased the level of PD-L1, and finally promoted the immune evasion of HCC.

## Discussion

The immunomodulatory role played by HSCs during HCC development has been widely discussed. It is known that HSCs can produce large amounts of ROS when activated, but little is known about their role. Based on the important impact of ROS in cancer development, we analyzed the role of ROS within HSCs in the immune evasion of HCC. Under oxidative stress conditions, the intracellular electron transport chain generates the first ROS-O_2_^•−^, which is a precursor of several ROS and has a remarkable influence in biological signal transduction. However, it is not clear about how O_2_^•−^ within HSCs affects downstream bioactive molecules in relation to immune evasion from HCC. Utilizing the probe TPH, we observed a positive correlation between the level of O_2_^•−^ in HSCs and immune evasion of HCC. This finding provides strong evidence that ROS in HSCs promotes immune evasion from HCC.

It has been reported that activated-HSCs are able to help tumor cells immune evasion by upregulate PD-L1, but its regulatory mechanism is unclear. Meanwhile, some researchers have found a link between ROS and PD-L1. Using fluorescence imaging, the elevated levels of O_2_^•−^ were found in cells and in vivo. This result prompted us to explore whether O_2_^•−^ within HSCs can regulate the expression of PD-L1 during oxidative stress. Therefore, we analyzed the relationship between O_2_^•−^ and PD-L1 in detail. The results of western blot experiments displayed that inducing O_2_^•−^ overproduction could increase the level of PD-L1. Meanwhile, excess O_2_^•−^ could significantly promote the growth of HCC in mice. On the contrary, the level of PD-L1 was significantly reduced and the growth of HCC was effectively delayed by decreased O_2_^•−^. In conclusion, we found that O_2_^•−^ within HSCs can upregulate intracellular PD-L1, which ultimately leads to immune evasion from HCC.

It was found that the expression of PD-L1 was regulated by CDK4. Numerous experiments have shown that excess O_2_^•−^ can damage DNA and disrupt protein structure, thus affecting the normal physiological functions of organisms. We were interested in whether O_2_^•−^ regulates CDK4 activity. The western blot experiments revealed that excess O_2_^•−^ could reduce the levels of molecules downstream of CDK4. In contrast, the phenomenon disappeared when O_2_^•−^ was removed. The above results suggest that excess O_2_^•−^ within HSCs can increase PD-L1 levels by decreasing CDK4 activity. The results of proteomics analysis experiments further indicated that excess O_2_^•−^ could oxidize amino acid residues in the CDK4 active site. These data convincingly suggest that O_2_^•−^ in HSC induces upregulation of PD-L1 levels by impairing CDK4 activity, and eventually lead to immune evasion. In conclusion, we provided a signaling pathway for the immune evasion of HCC mediated by O_2_^•−^ within HSCs.

**Fig. 9:**
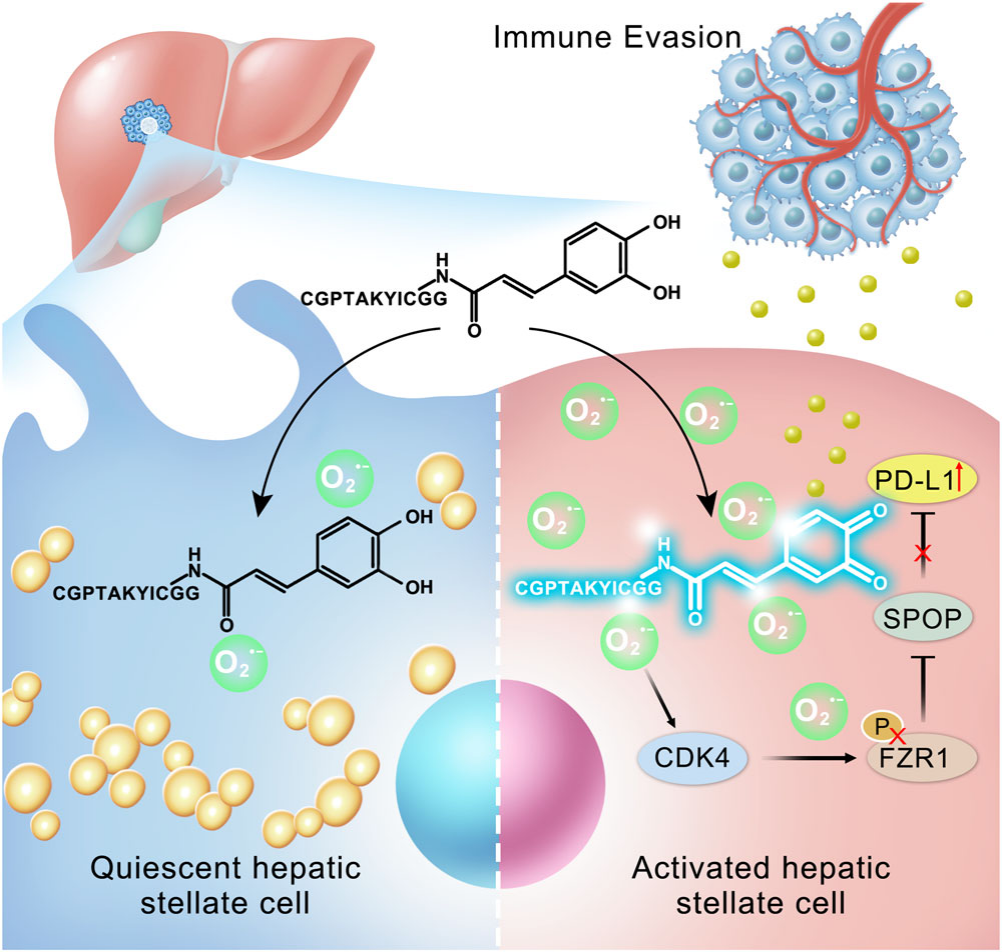
Signal pathway of HCC immune evasion mediated by O_2_^•−^ in HSCs. HSCs produce large amounts of O_2_^•−^ upon activation. Excessive O_2_^•−^ damages the active region of CDK, preventing CDK4 from phosphorylating downstream FZR1. Unphosphorylated FZR1 prevents SPOP from degrading PD-L1, which raises PD-L1 levels.

In order to explore the role of O_2_^•−^ in HSCs in the immune evasion of HCC, we developed a two-photon fluorescence probe TPH for specific imaging detection of O_2_^•−^. Using TPH, we observed the increase of O_2_^•−^ during HSCs activation in cells and in vivo. Notably, we found that the immunosuppressive ability of the HCC microenvironment was correlated with the activation status of HSCs. Further studies indicated that the excess O_2_^•−^ generated HSCs modified the functional regions of CDK4, resulting in the inactivation of CDK4. Subsequently, the phosphorylation of FZR1 decreased, and caused to more SPOP degraded. The decrease of SPOP led to the increase of PD-L1 in HCC mice, conclusively inducing immune evasion (Fig. 9). This work provided a molecular mechanism by which O_2_^•−^ in HSCs mediated immune evasion from HCC. We propose that this work can provide a supplement to understanding the occurrence and development of HCC. Meanwhile, it is expected to provide new ideas for immunotherapy methods for HCC.

## Methods

### Ethical statement

All animal care and experimental protocols complied with the Animal Management Rules of the Ministry of Health of the People’s Republic of China and were approved by the Animal Care Committee of Shandong Normal University (AEECSDNU2022023).

### Measurement of two-photon cross sections

The two-photon absorption cross sections *δ* were determined using the femtosecond (fs) fluorescence measurement technique. TPH was dissolved in cell lysis buffer at a concentration of 40 μM, and then the TP-excited fluorescence spectra were measured at 800 nm. Fluorescein (1.0 × 10^−4^ M, pH = 11) whose TP properties have been well characterized in the literature, was used as the reference. The value of *δ* was calculated using the following equation.

1 $${\delta }_{{{{{\rm{s}}}}}}={\delta }_{{{{{\rm{r}}}}}}\frac{{\varPhi }_{{{{{\rm{r}}}}}}}{{\varPhi }_{{{{{\rm{s}}}}}}}\frac{{C}_{{{{{\rm{r}}}}}}}{{C}_{{{{{\rm{s}}}}}}}\frac{{n}_{{{{{\rm{r}}}}}}}{{n}_{{{{{\rm{s}}}}}}}\frac{{F}_{{{{{\rm{s}}}}}}}{{F}_{{{{{\rm{r}}}}}}}$$

The subscripts *s* and *r* refer to the sample and the reference material, respectively. *δ* is the TP absorption cross-sectional value, *C* is the concentration of the solution, *n* is the refractive index of the solution, *F* is the TP-excited fluorescence intensity, and *Φ* is the fluorescence quantum yield.

### Cell culture

LX-2 cells were purchased from Procell Life Science & Technology Co., Ltd. (Wuhan, China), and cultured in DMEM medium with 10% fetal bovine serum, 1% streptomycin, and 1% penicillin. The cells were placed in an MCO-15AC incubator (Sanyo, Tokyo, Japan) with culture parameters of 37 °C, 5% CO_2_, and 95% air. Two days before the cell imaging experiment, we digested the cells with trypsin and put an appropriate amount of cells into the culture dish for the convenience of the subsequent experimental operation.

### Cytotoxicity assays

The cytotoxicity of 2-(2-methoxy-4-nitrobenzene)-3-(4-nitrobenzene)-5-(2,4-disulfonybenzene)-2h-tetrazole monosodium salt (CCK8) was tested. LX-2 cells were inoculated on 96-well plates with a concentration of 1 × 10^5^ cell pore^−1^ and placed in an incubator for 24 h. Then, cells were incubated with different concentrations of probes for 12 h. The cells were rinsed with PBS, followed by 10 µL CCK8 solution and 90 µL medium were added to per well. After 1 h, the absorbance at 450 nm was measured by enzyme-labeled instrument, and the survival rate of cells was calculated.

### Mice

All male C57 mice (age: 4–6 weeks; average weight: 20 ± 2 g) were purchased from Jinan Pengyue Experimental Animal Breeding Company Limited. The purchased 4–6 weeks C57BL/6 mice were placed in the animal house with 12 h light to ensure adequate water and food for the mice. After a week of adaptation, the mice were randomly grouped to construct mouse models of HSCs with different activation degrees and liver cancer models of HSCs with different activation degrees, respectively.

### The mouse models with various levels of aHSCs

Mice were intraperitoneally injected with olive oil or CCl_4_ solution (1.0 mL kg^−1^) twice a week for 2, 4, and 6 weeks, respectively. Mice in the treatment group were injected with D-pen (100 mg kg^−1^) or vitamin E (100 mg kg^−1^) twice a week for 2 weeks after 6 weeks of CCl_4_ solution.

### The orthotopic mouse models of HCC containing quiescent-HSCs or aHSCs

Mice were intraperitoneally injected with olive oil or CCl_4_ solution (1.0 mL kg^−1^) twice a week for 6 weeks, and then the orthotopic mouse models of HCC were constructed. Mice in the treatment group were injected with D-pen (100 mg kg^−1^) or VE (100 mg kg^−1^) for 2 weeks after 6 weeks of CCl_4_ solution, and then the orthotopic mouse models of HCC were constructed.

### Fluorescence imaging experiments in vivo

In vivo imaging experiments, TPH was first injected intraperitoneally into model mice with 0.62 mg kg^−1^. After 30 min, the mice were injected intraperitoneally to numb them, and the mice were opened. The fluorescence imaging was performed using a two-photon fluorescence imaging microscope at an excitation wavelength of 800 nm.

### Proteomic analysis

The CDK4 was dissolved in the appropriate buffer (50 mM Tris, pH = 8.0). Then, 1 mM superoxide anion (O_2_^•−^) was added, and incubated at 37 °C for 2 h. CDK4 was trypsin digested, and the peptides were isolated from the hydrolysate by solid phase extraction on the C-18 column. Proteomic analysis was performed by LC–MS/MS.

### Statistics and reproducibility

The data were analyzed using Origin Pro 2021 (64-bit). Student’s *t* test was used to evaluate the differences between the data. *P* values < 0.05 were considered statistically significant. It was defined as *P < 0.05, **P < 0.01, and ***P < 0.001.

### Reporting summary

Further information on research design is available in the Nature Portfolio Reporting Summary linked to this article.

### Supplementary information

Supporting information

Description of Additional Supplementary Files

Supplementary Data 1

Reporting Summary

## Data Availability

All the data generated in this study are provided in the Supplementary Information/Source Data file. There are no restrictions on data availability in the current work. The mass spectrometry proteomics data have been deposited to the ProteomeXchange Consortium (http://proteomecentral.proteomexchange.org) via the iProX partner repository^34,35^ with the dataset identifier PXD051328.
