# Supplementary material for: Fluorescence imaging sheds light on the immune evasion mechanisms of hepatic stellate cells mediated by superoxide anion
Source: Commun Biol. 2024 May 10;7:558. doi: 10.1038/s42003-024-06245-y (PMC11087649; doi:10.1038/s42003-024-06245-y)
Supplement: Supplementary file 1 — Supporting information [file 42003_2024_6245_MOESM1_ESM.docx]

Supporting Information

Fluorescence imaging sheds light on the immune evasion mechanisms of hepatic stellate cells mediated by superoxide anion

Yuantao Mao^1, ‡^, Chuanchen Wu^1,^ ^‡^, Xin Wang^1, *^, Fanghui Zhang^1^, Xinru Qi^1^, Xia Li^2, *^, Ping Li^1, *^& Bo Tang^1, 3,^ ^*^

^1^ College of Chemistry, Chemical Engineering and Materials Science, Collaborative Innovation Center of Functionalized Probes for Chemical Imaging in Universities of Shandong, Key Laboratory of Molecular and Nano Probes, Ministry of Education, Institute of Biomedical Sciences, Shandong Normal University, Jinan 250014, P. R. China.

^2^ Innovative Institute of Chinese Medicine and Pharmacy, Shandong University of Traditional Chinese Medicine, Jinan, China.

^3^ Laoshan Laboratory, 168 Wenhai Middle Rd, Aoshanwei Jimo, Qingdao 266237, Shandong, China.

Corresponding Authors: *E-mail: tangb@sdnu.edu.cn, *E-mail: [lip@sdnu.edu.cn](mailto:lip@sdnu.edu.cn), *E-mail: [60230033@sdutcm.edu.cn](mailto:lixia@sdutcm.edu.cn), *E-mail: xinwang@sdnu.edu.cn.

**Experimental Procedures**

**Materials and reagents**

All reagents used in the experiment were analytically pure concentrations and were not treated before use. Dimethyl sulfoxide (DMSO), sodium nitrite (NaNO2), hydrogen peroxide (H_2_O_2_), and olive oil were all purchased from Sinopyma Chemical Reagent Co., Ltd. Carbon tetrachloride (CCl_4_) was purchased from Shanghai Maclin Biochemical Technology Co., Ltd. Caffeic acid, tocopherol (VE), Tiron, and D-penicillamine were purchased from Shanghai Aladdin Biochemical Technology Co., Ltd. Dimethylestradiol (2-Me), 3- (4, 5-dimethylthiazole-2-yl) -2, 5-diphenyltetrazole bromide (CCK-8) were purchased from Sigma-Aldrich. TGF-β1 was purchased from Beijing Yiqiao Shenzhou Technology Co., Ltd.

CCl_4_ solution: The CCl_4_ reagent and olive oil in accordance with the ratio of 1:4 miscibility.

**Results**


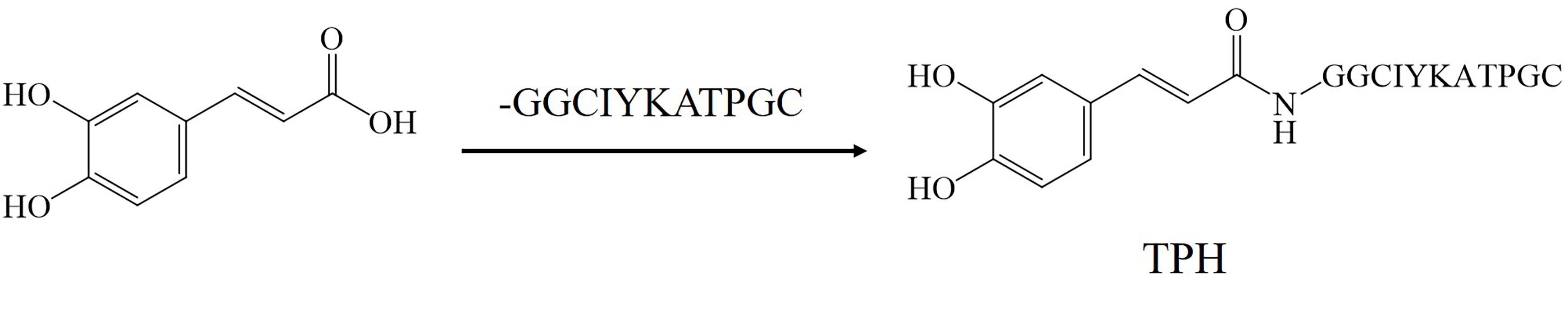


Supplementary Figure 1. Synthesis of TPH, which was provided by GL Biochem Ltd. (Shanghai China).


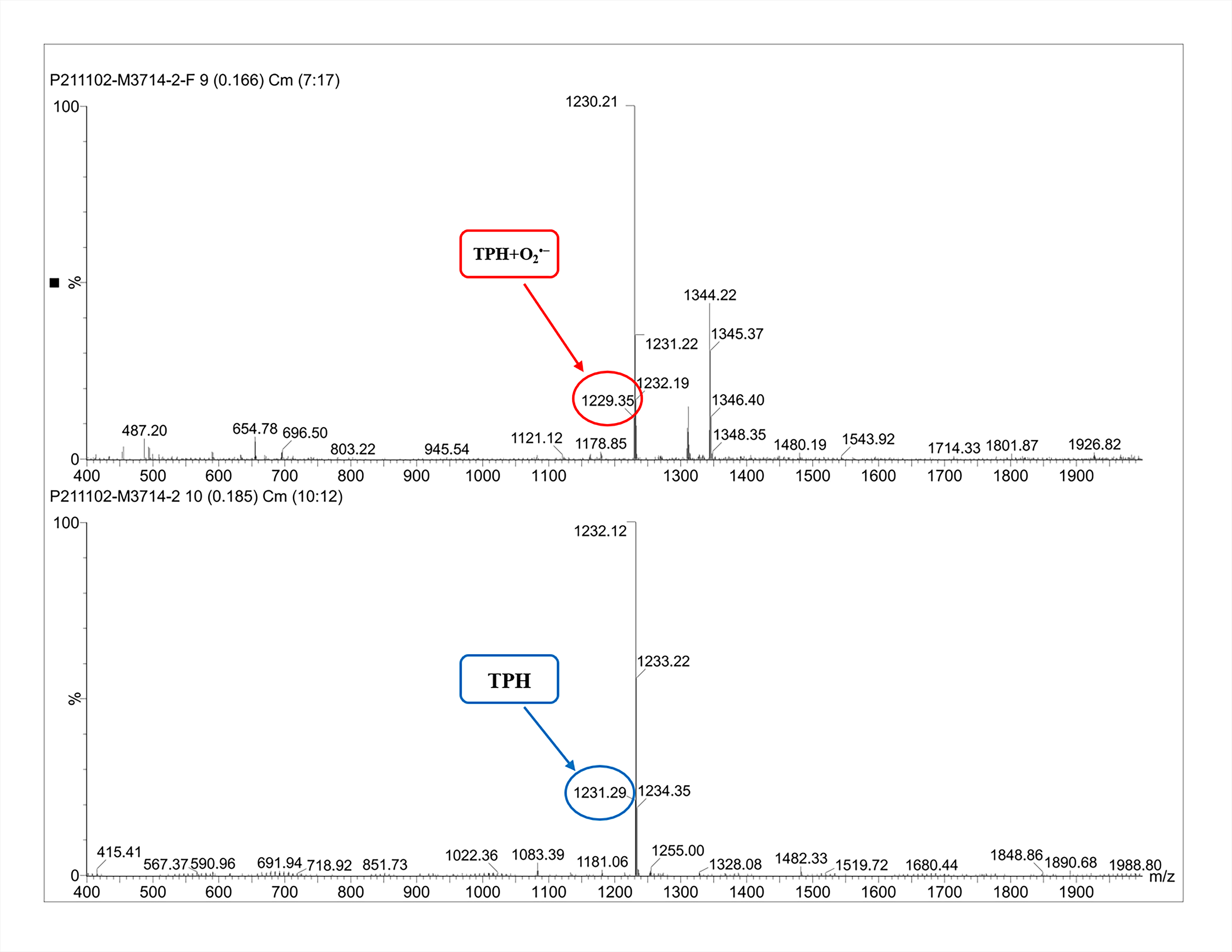


Supplementary Figure 2. HRMS before and after the reaction of TPH with O_2_^•−^.


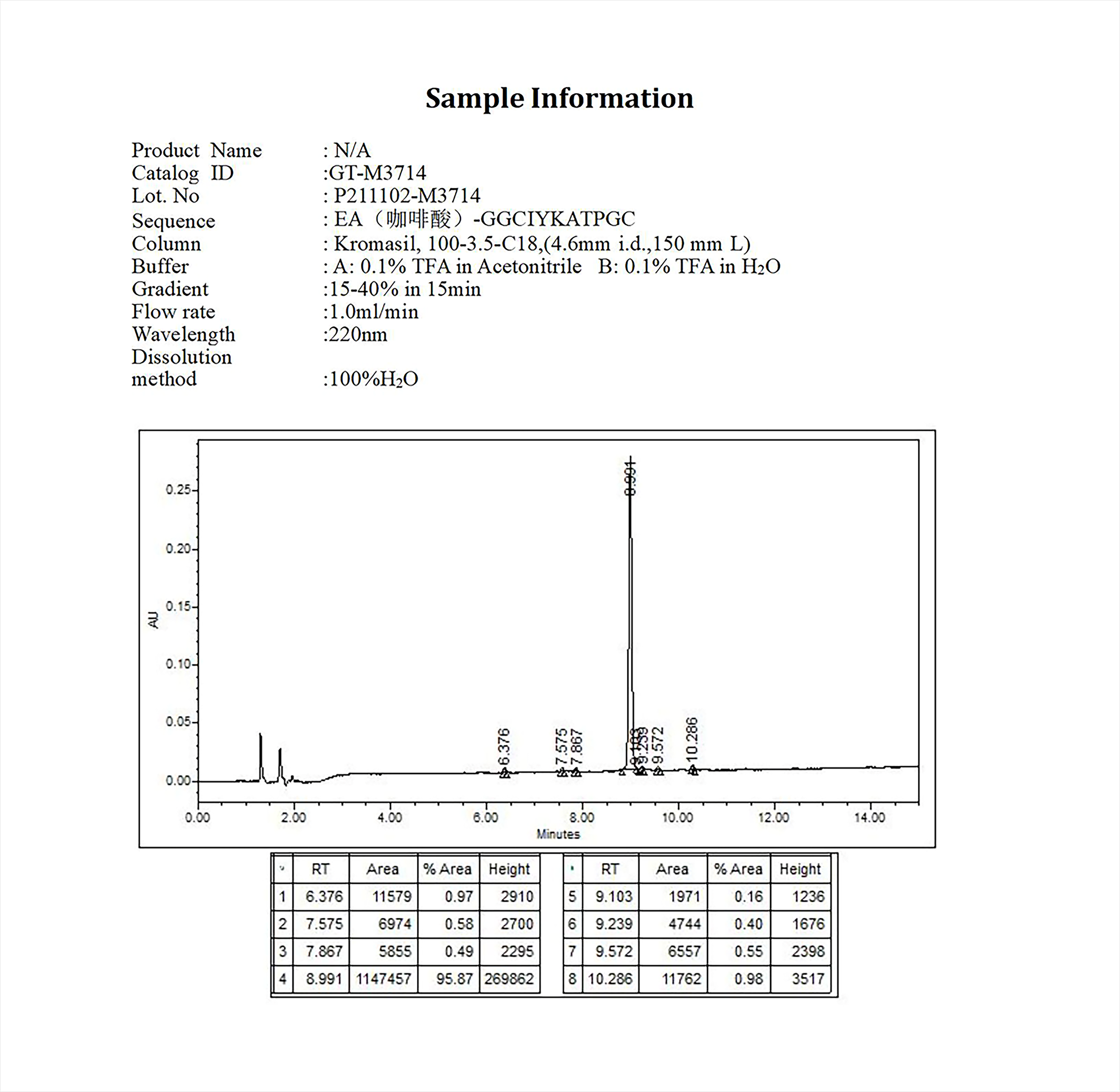


Supplementary Figure 3. The High Performance Liquid Chromatography (HPLC) experiment of TPH.


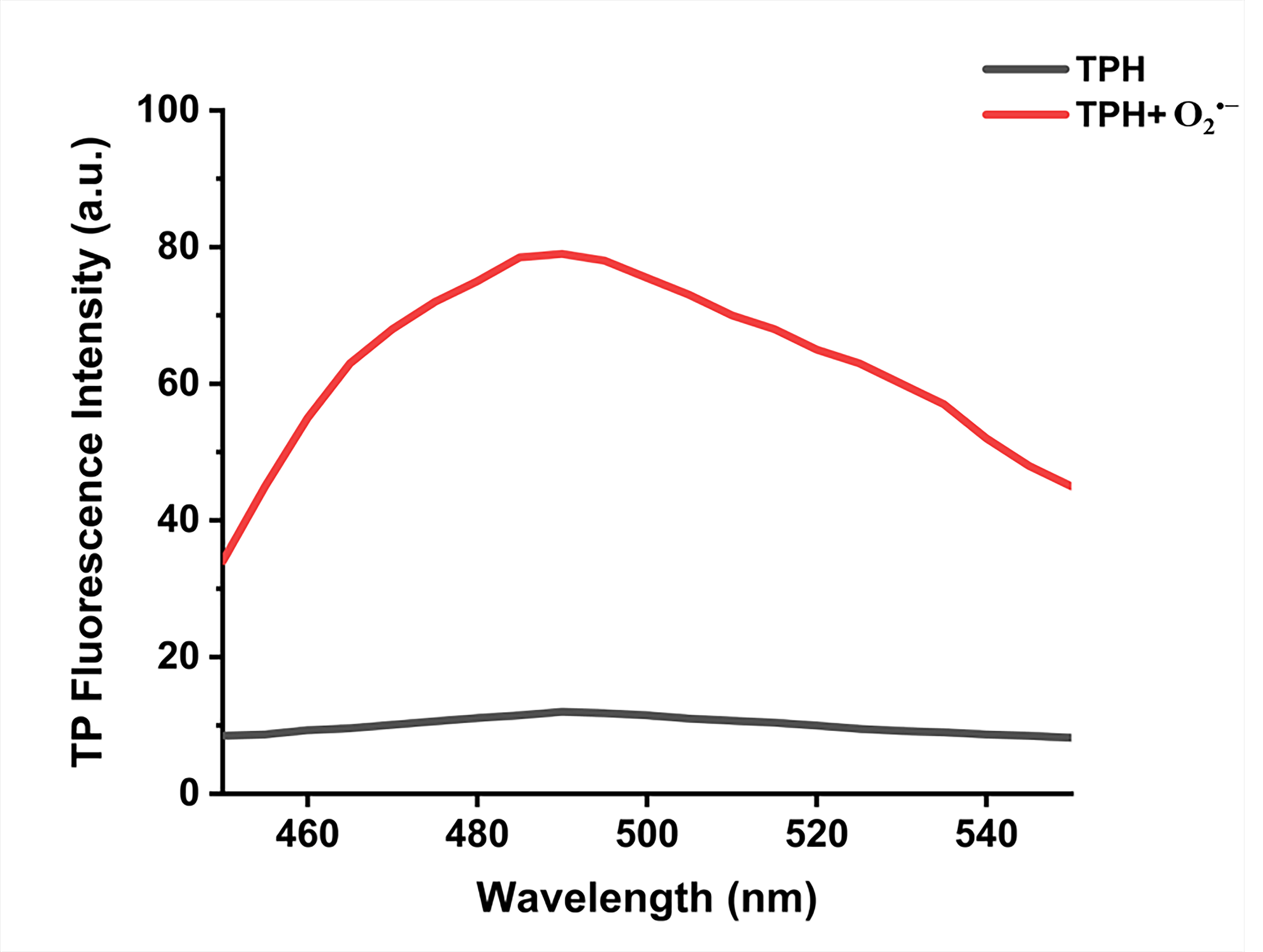


Supplementary Figure 4. Two-photon fluorescence response of TPH to O_2_^•−^. Two-photon fluorescence spectra of 40 μM TPH (black line) alone and after the addition of 20 μM O_2_^•−^ (red line). λex= 800 nm.


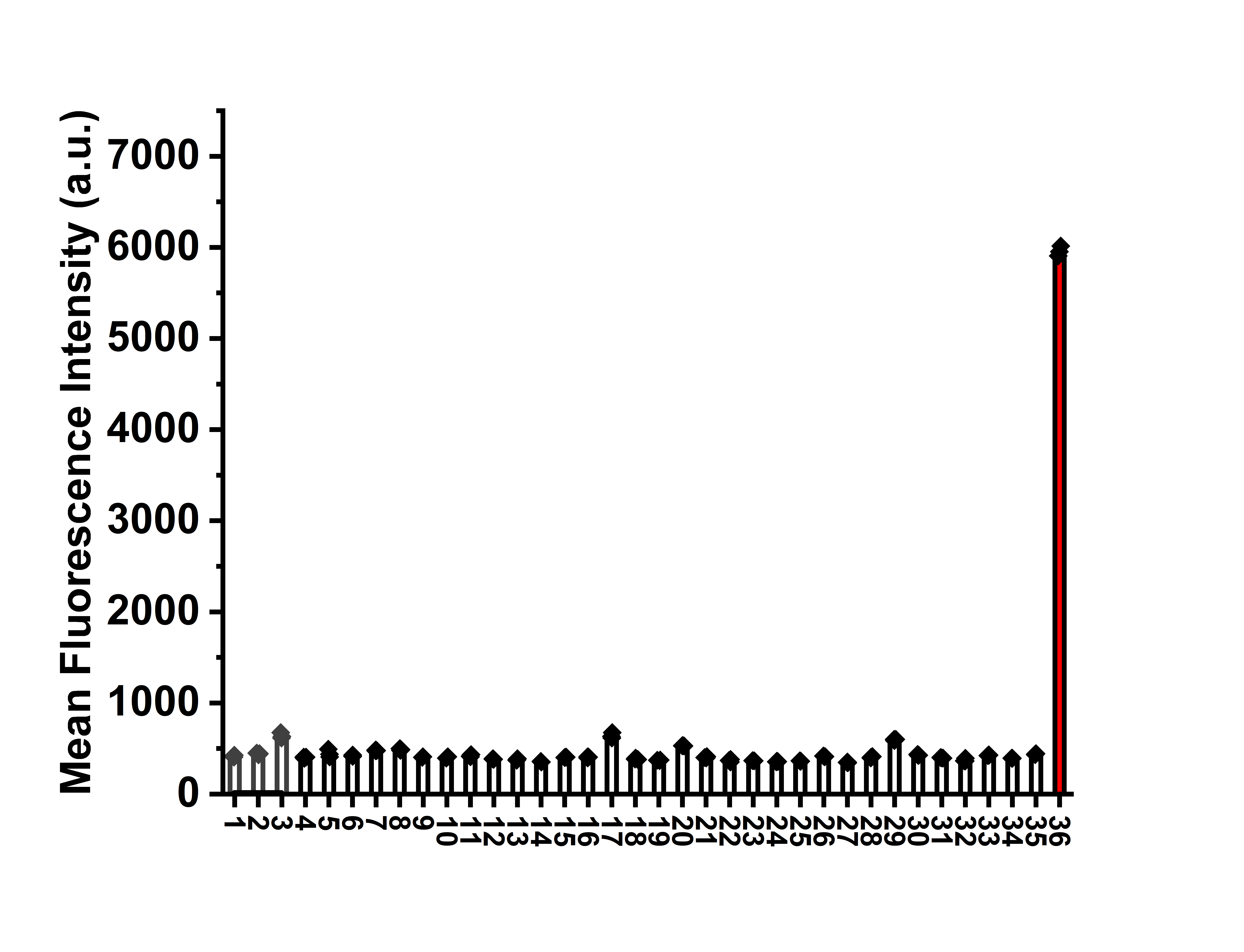


Supplementary Figure 5. TPH selectivity experiment. Fluorescence response spectra of 40 μM TCP with ROS, RON, metal ions and amino acids (cell extract was used as buffer, pH = 7.4, λ ex= 370 nm, λ em = 490 nm). Interfering substances: (1-2) 100 μM **^.^**OH, NO, (3) 100 nM ONOO^-^, (4-6) 100 μM ^1^O_2_, TBPH, NaClO, (7) 10 mM H_2_O_2_, (8-9) 100 μM Cu^2+^, Fe^3+^, (10-11) 10 mM Na^+^, K^+^, (12-16) 100 μM Ni^2+^, Mg^2+^, Ca^2+^, Zn^2+^, Mn^2+^, (17) 5 μM Al^3+^, (18) 100 μM Fe^2+^, (19) 100 μM Ala, (20) 50μM Arg, (19, 21-35) 100 μM Ala, Asn, Asp, Gln, Gly, GSH, His, Ile, L-Cys, Lys, Met, Phe, Pro, Ser, Thr, Val, (36) 20 μM O_2_^•−^**.**


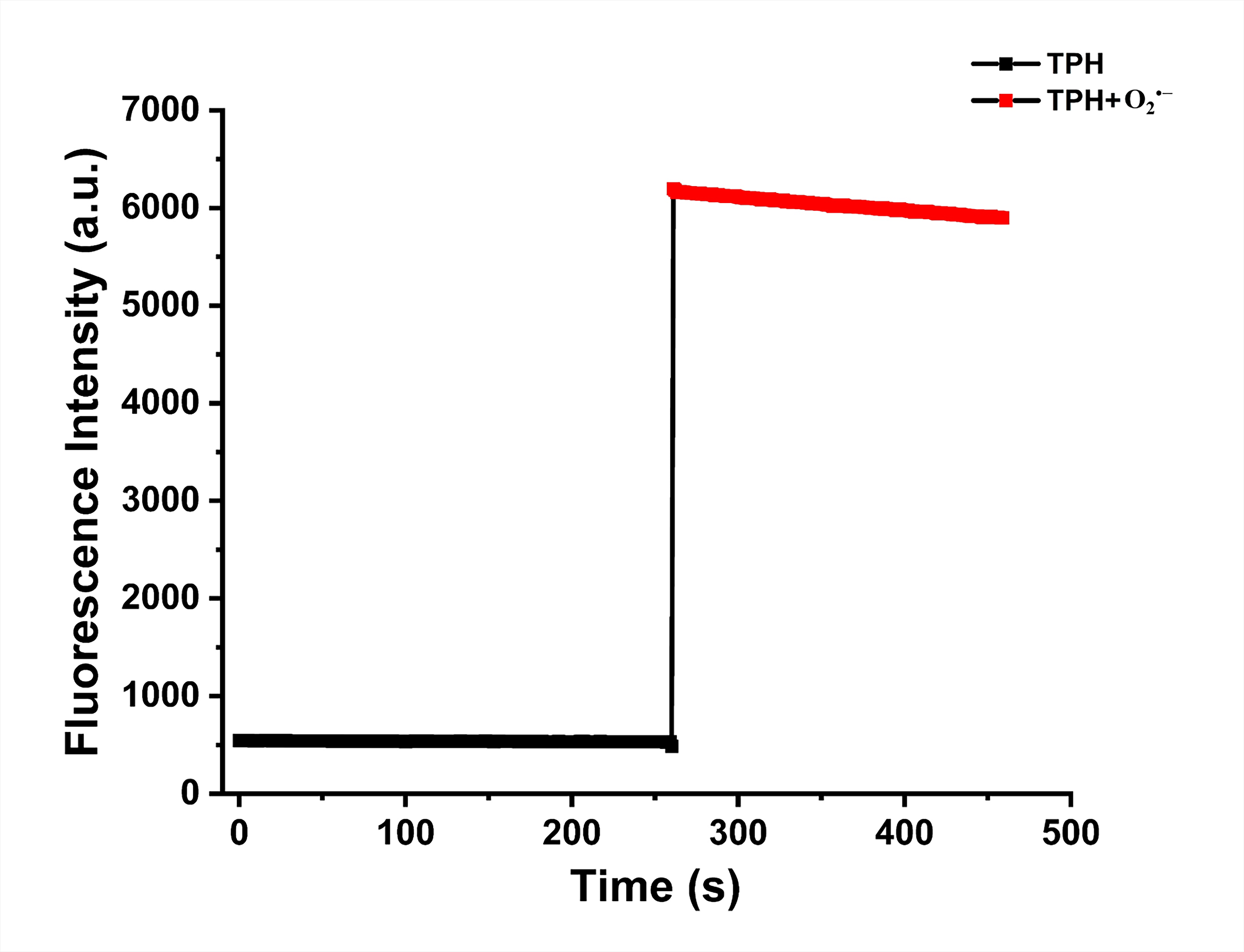


Supplementary Figure 6. Response time experiment. The instantaneous response experiment of 40 µM TPH before (black) and after (red) the addition of O_2_^•−^ (20 μM) with λex = 370 nm and λem = 490 nm.


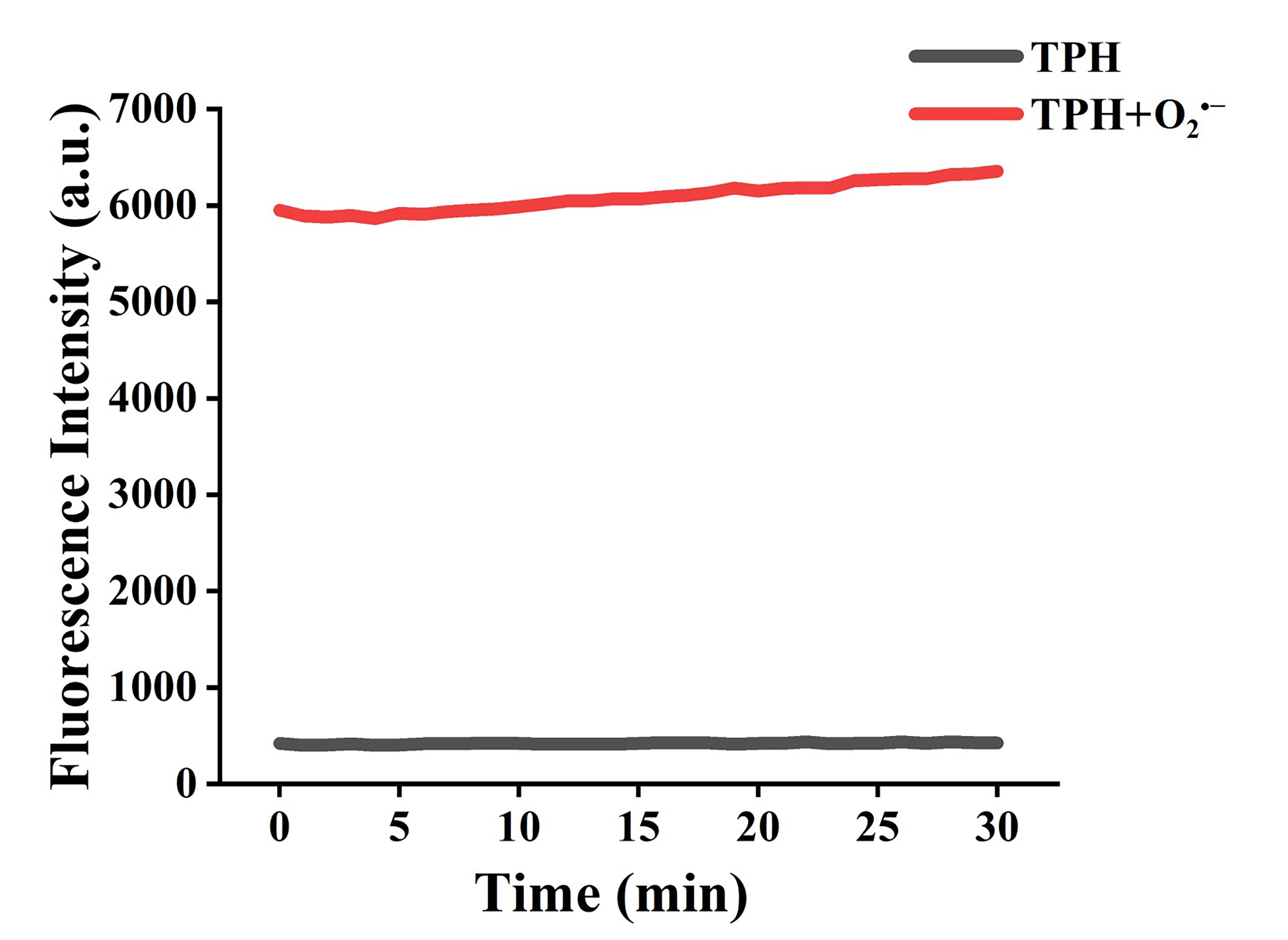


Supplementary Figure 7. Photostability before and after TPH reaction. The fluorescence intensity of 40 μM TPH before (black) and after (red) the addition of 20 μM O_2_^•−^ (cell extract was used as buffer, pH = 7.4, λ ex= 370 nm, λ em = 490 nm).


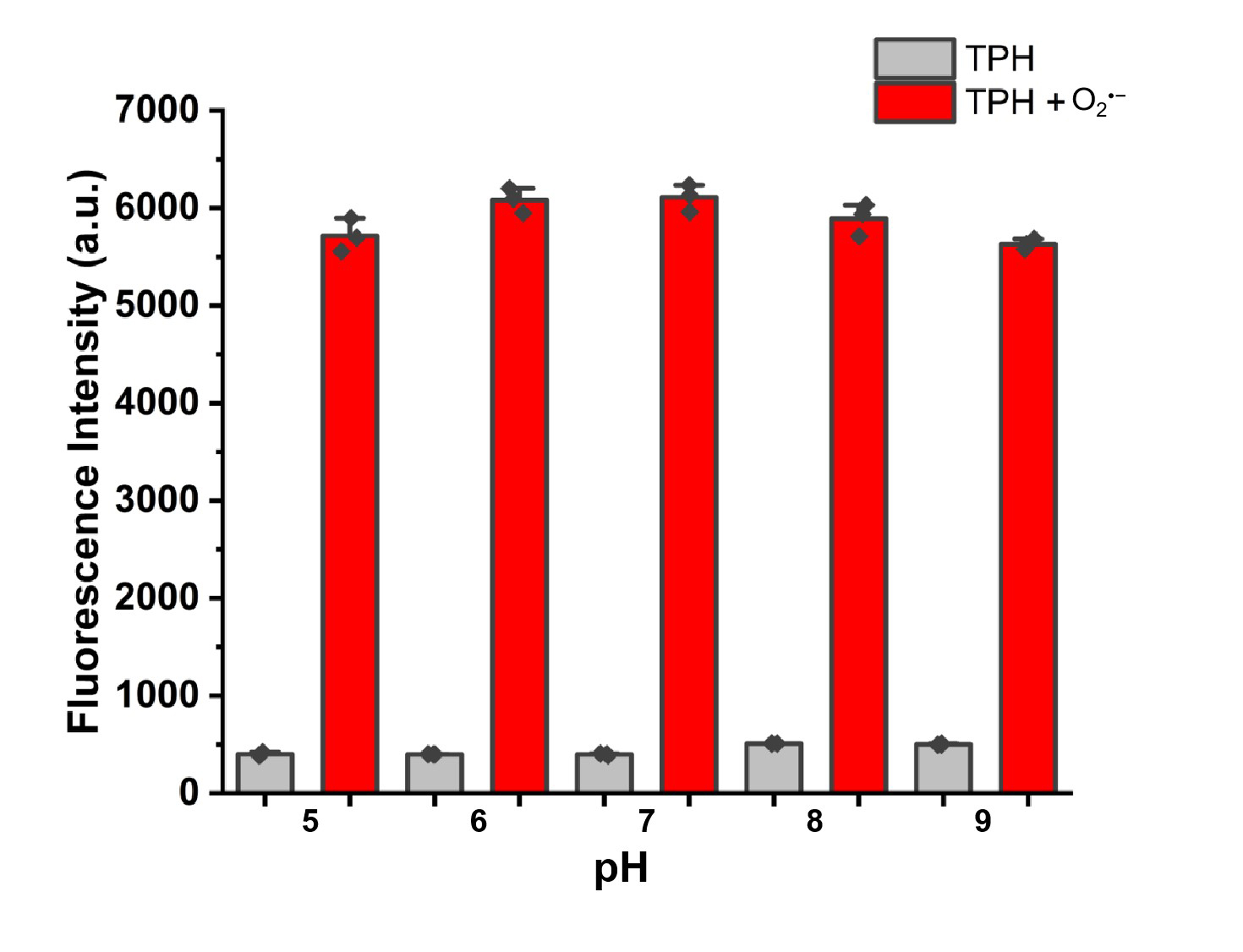


Supplementary Figure 8. Fluorescence intensities of 40 μM TPH (black) and after the addition of 20 μM O_2_^•−^ (red) in the solution with various pH values.


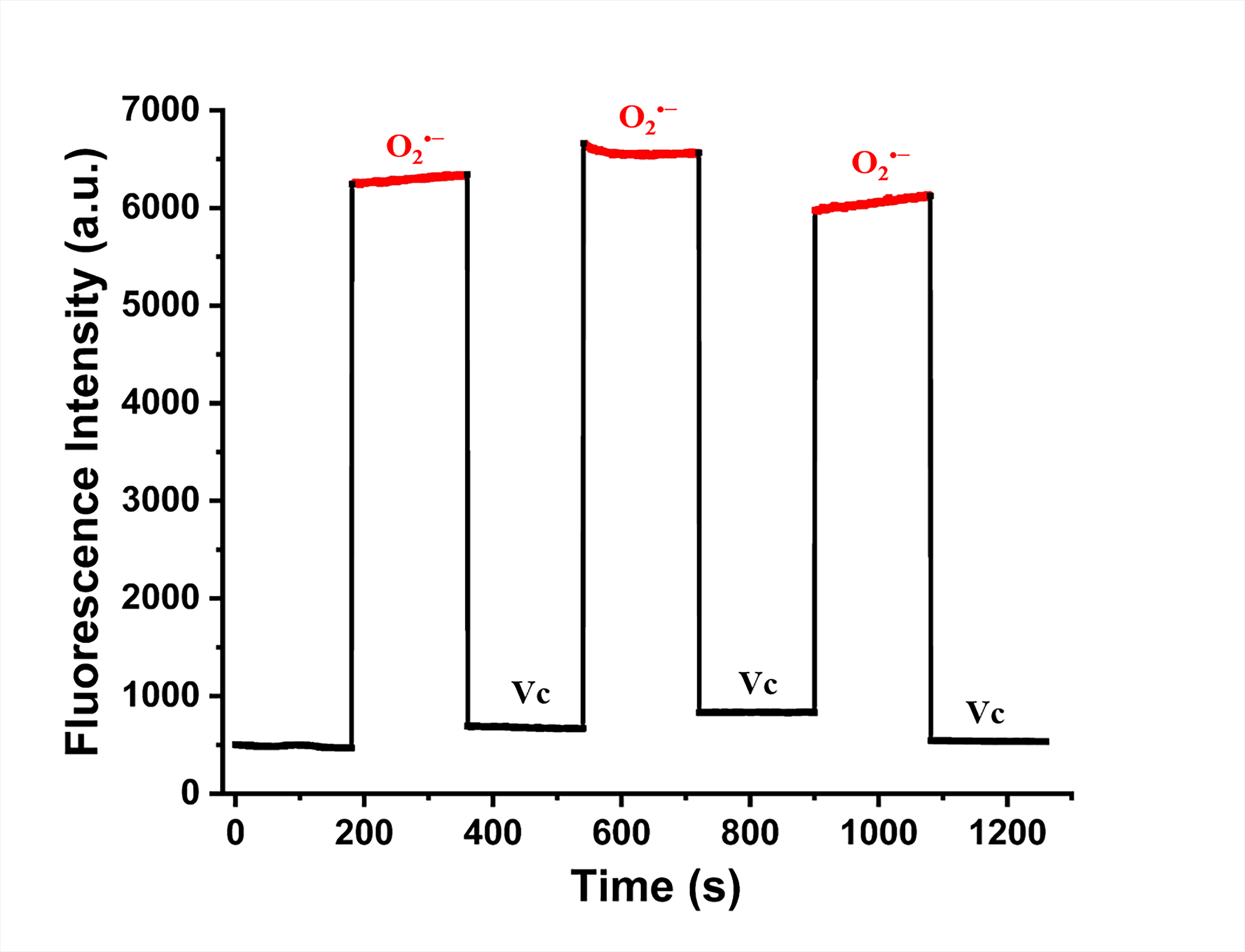


Supplementary Figure 9. Reversibility of 40 μM TPH fluorescence in the presence of alternating treatments with 20 μM O_2_^•−^ and 1 mM ascorbic acid (Vc).


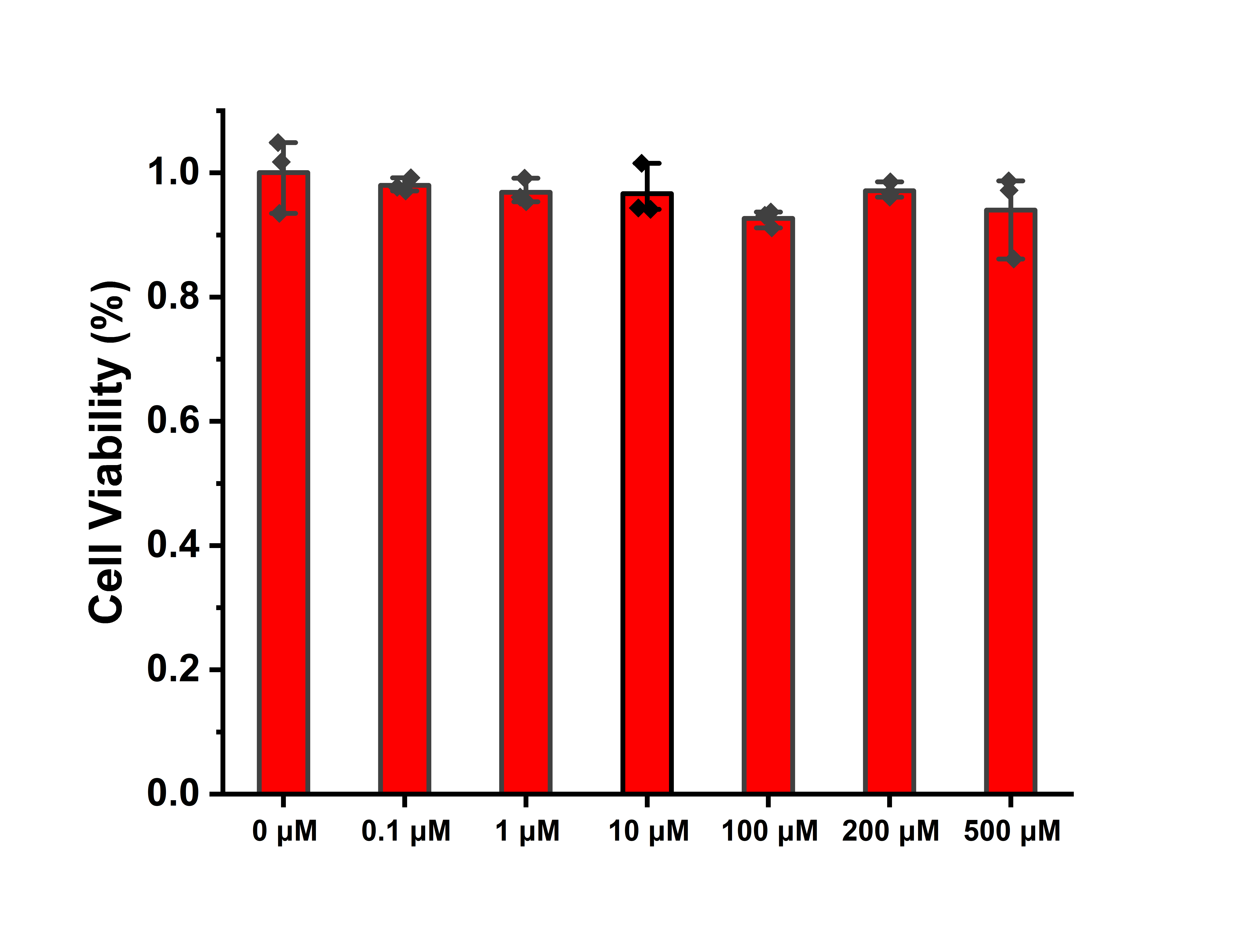


Supplementary Figure 10. CCK8 experiment. LX-2 cells were incubated with different concentrations of TPH for 12 h, and CCK8 solution (100 μL ml^-1^ DMEM) was added. Absorbance at 450 nm was measured 1 h later.


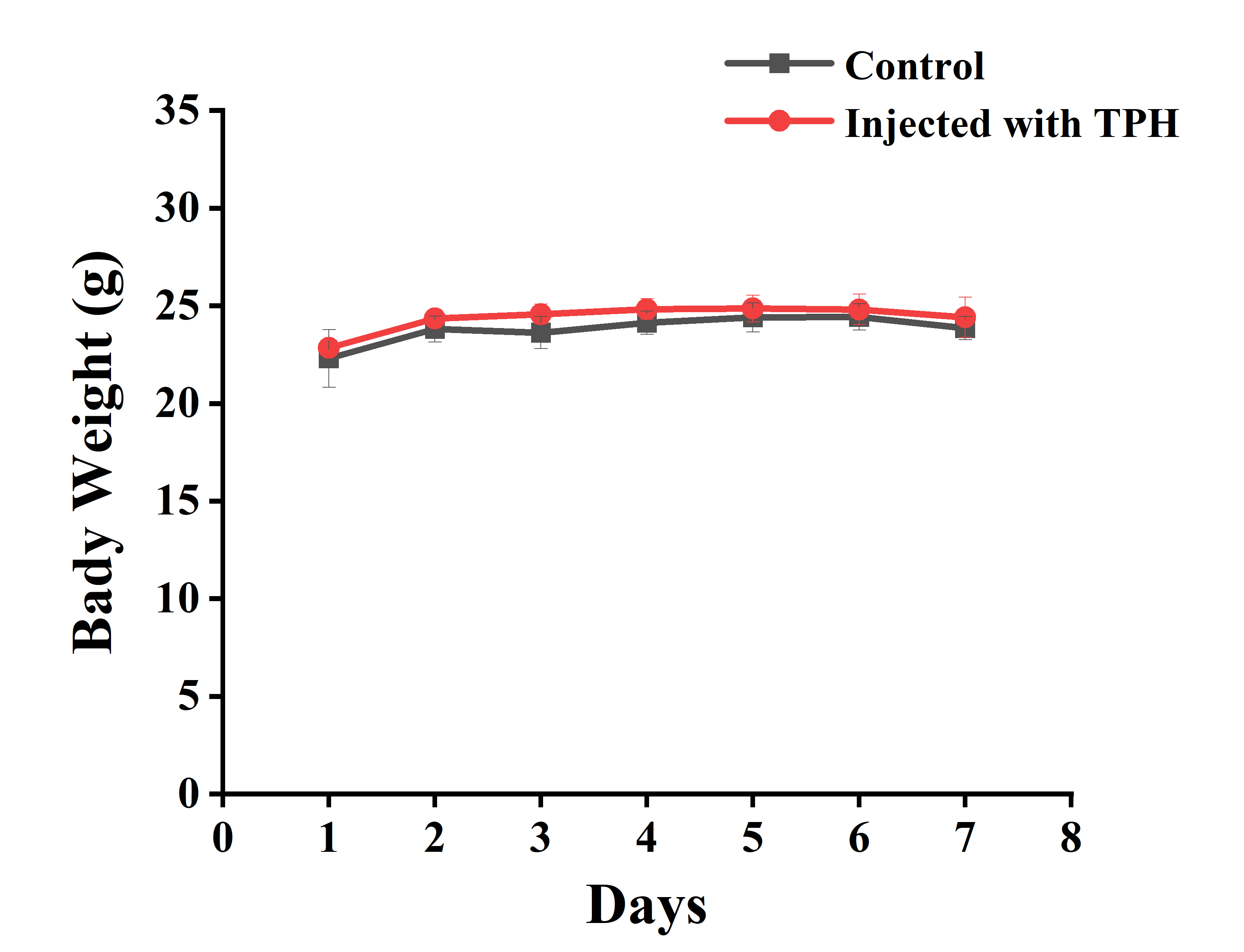


Supplementary Figure 11. The biocompatibility of TPH. Mice were randomly divided into a normal group and an experimental group, and intraperitoneally injected with 0.9% NaCl solution or 5 mM TPH, respectively. The body weights of mice were recorded for a week. Black line: mice injected with 0.9 % NaCl aqueous solution. Red line: mice injected with 5 mM TPH.


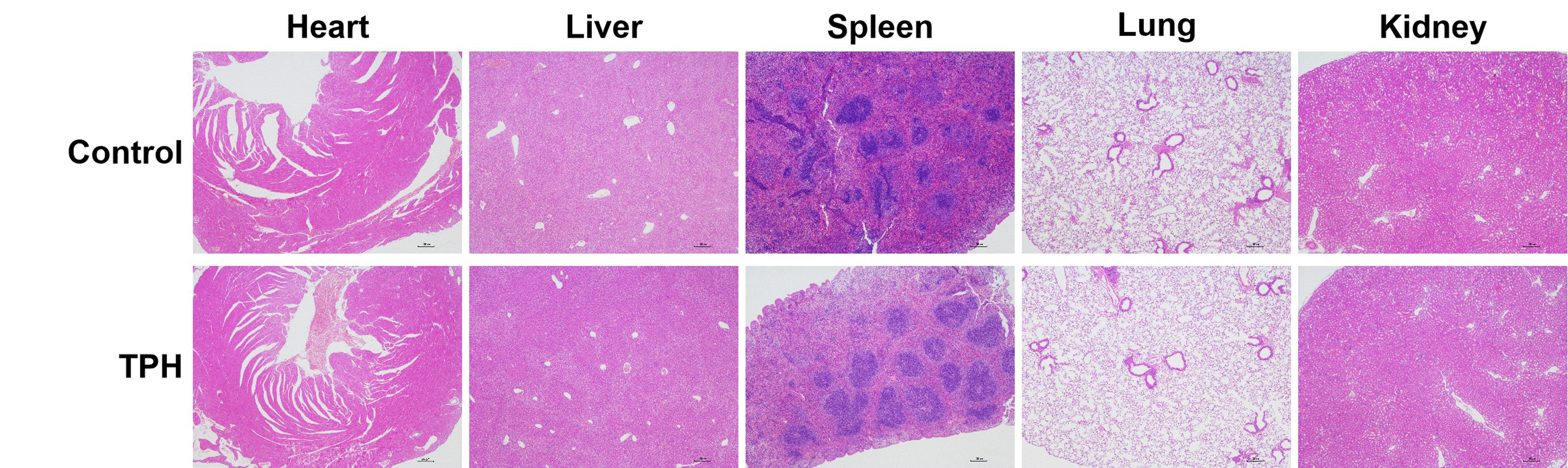


Supplementary Figure 12. Hematoxylin and Eosin (H&E) staining of major organs in mice. Control: mice intraperitoneally injected with normal saline. TPH: mice intraperitoneally injected with 5mM TPH. From left to right were the heart, liver, spleen, lung, and kidney. Scale bar = 200 μm.


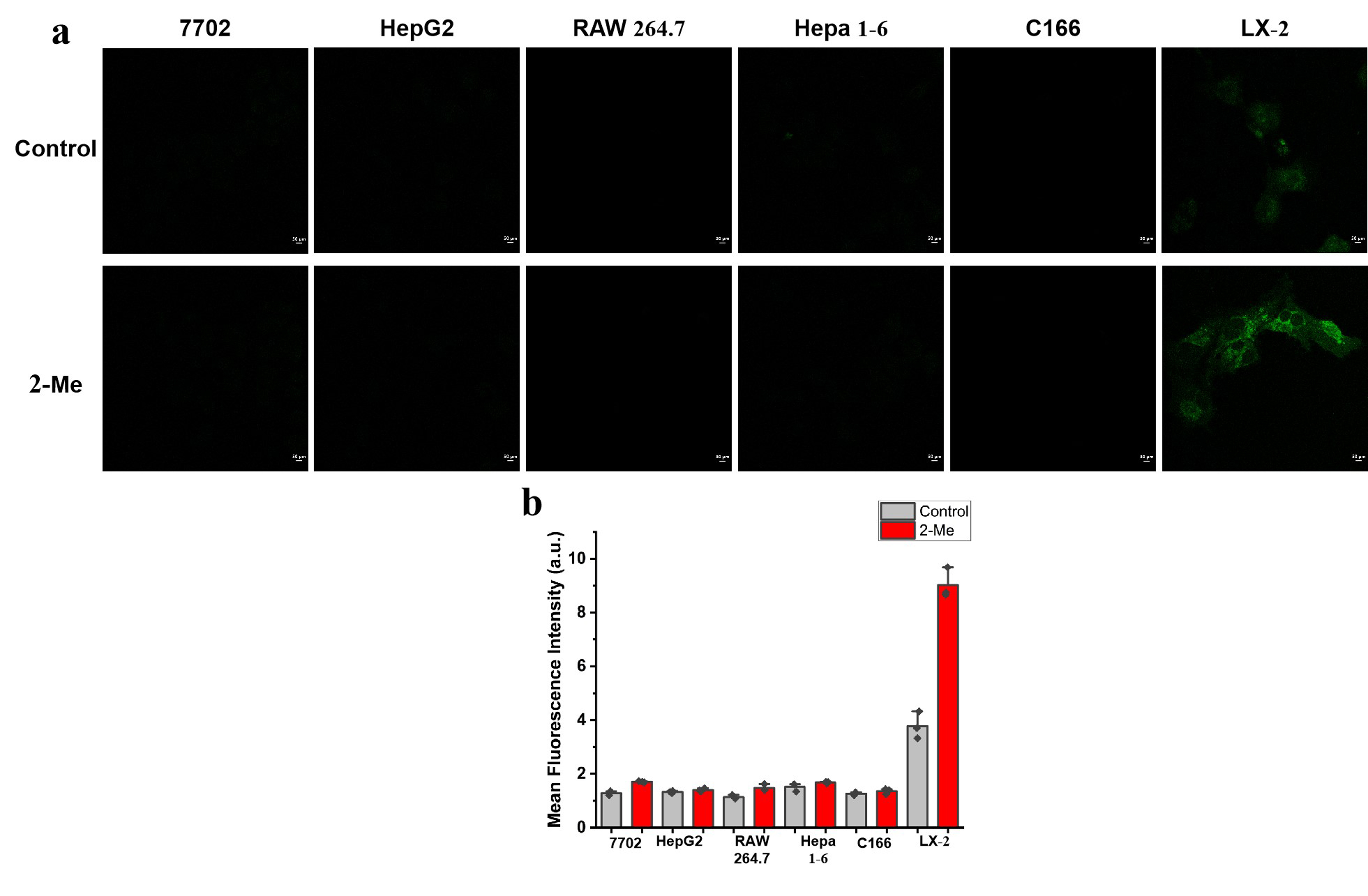


Supplementary Figure 13. Cell selectivity experiments. (A) O_2_^•−^ fluorescence imaging experiments of different cells. Control: TPH (50 μM) for 30 min. 2-Me: 2-Me (0.1 µg mL^-1^) for 15 min, and TPH (50 μM) for 30 min. 7702: human normal liver epithelial cells, HepG2: human hepatocellular carcinomas cells, RAW264.7: monocyte-macrophage cells, Hepa1-6: mouse hepatocellular carcinoma cells, C166: mouse vascular endothelial cells, LX-2: hepatic stellate cells. (B) Fluorescence intensities of A. Scale bar = 50 μm. The data were expressed as mean ± SD, n = 3.


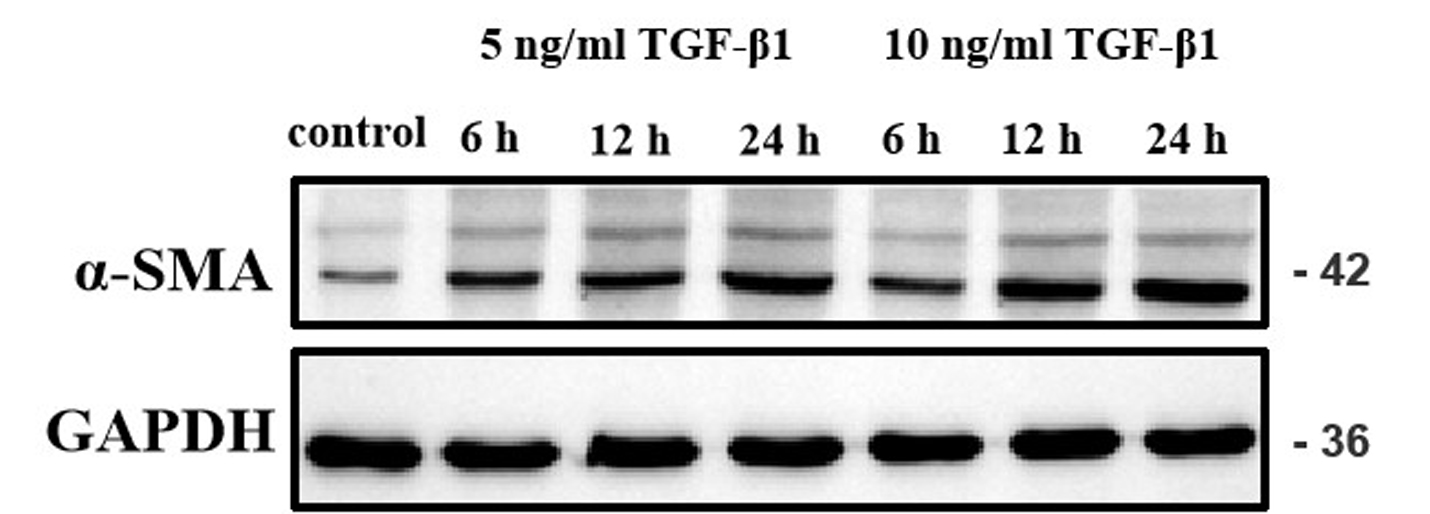


Supplementary Figure 14. The α-SMA Western blotting experiment of LX-2 cells with varying activation degrees. From left to right, the incubation conditions were control, 5 ng mL^-1^ TGF-β1 6 h, 5 ngmL^-1^ TGF-β1 12 h, 5 ng mL^-1^ TGF-β1 24 h, 10 ng mL^-1^ TGF-β1 6 h, 10 ng mL^-1^ TGF-β1 12 h, 10 ng mL^-1^ TGF-β1 24 h.


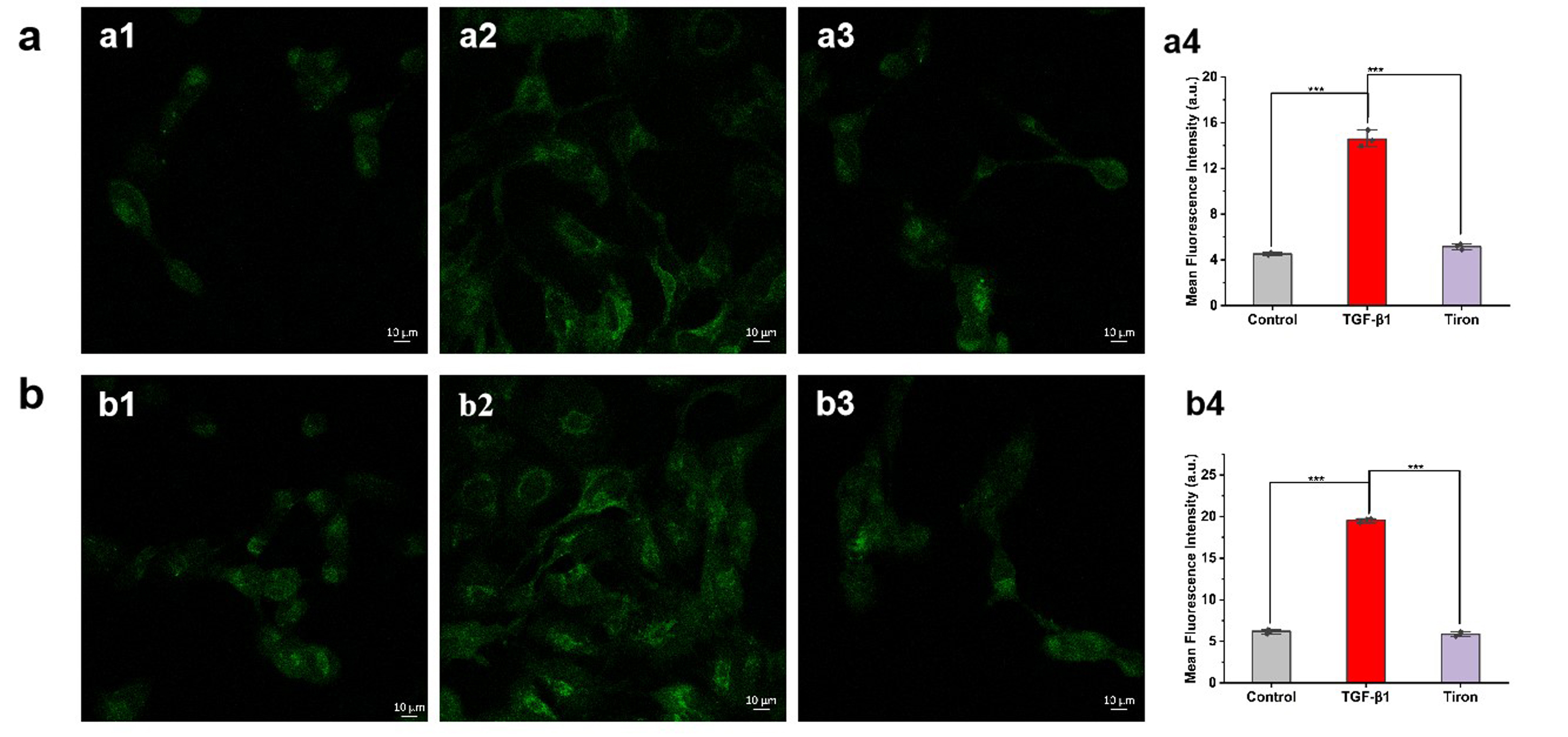


Supplementary Figure 15. O_2_^•−^ fluorescence imaging of LX-2 cells. a: O_2_^•−^ fluorescence imaging of LX-2 cells by one-photon excitation. λex = 405 nm and λem = 450-550 nm. b: O_2_^•−^ fluorescence imaging of LX-2 cells by two-photon excitation. λex = 740 nm and λem = 450-550 nm. (a1, b1): control; (a2, b2): TGF-β1 (5 ng mL^-1^) was incubated for 12 h; (a3, b3): TGF-β1 was incubated and Tiron (10 µM) was added for 30 min. (a4, b4): Fluorescence intensities of A and B. Scale bar = 10 μm.The data were expressed as mean ± SD, n = 3. ***P < 0.001 compared to the control group.


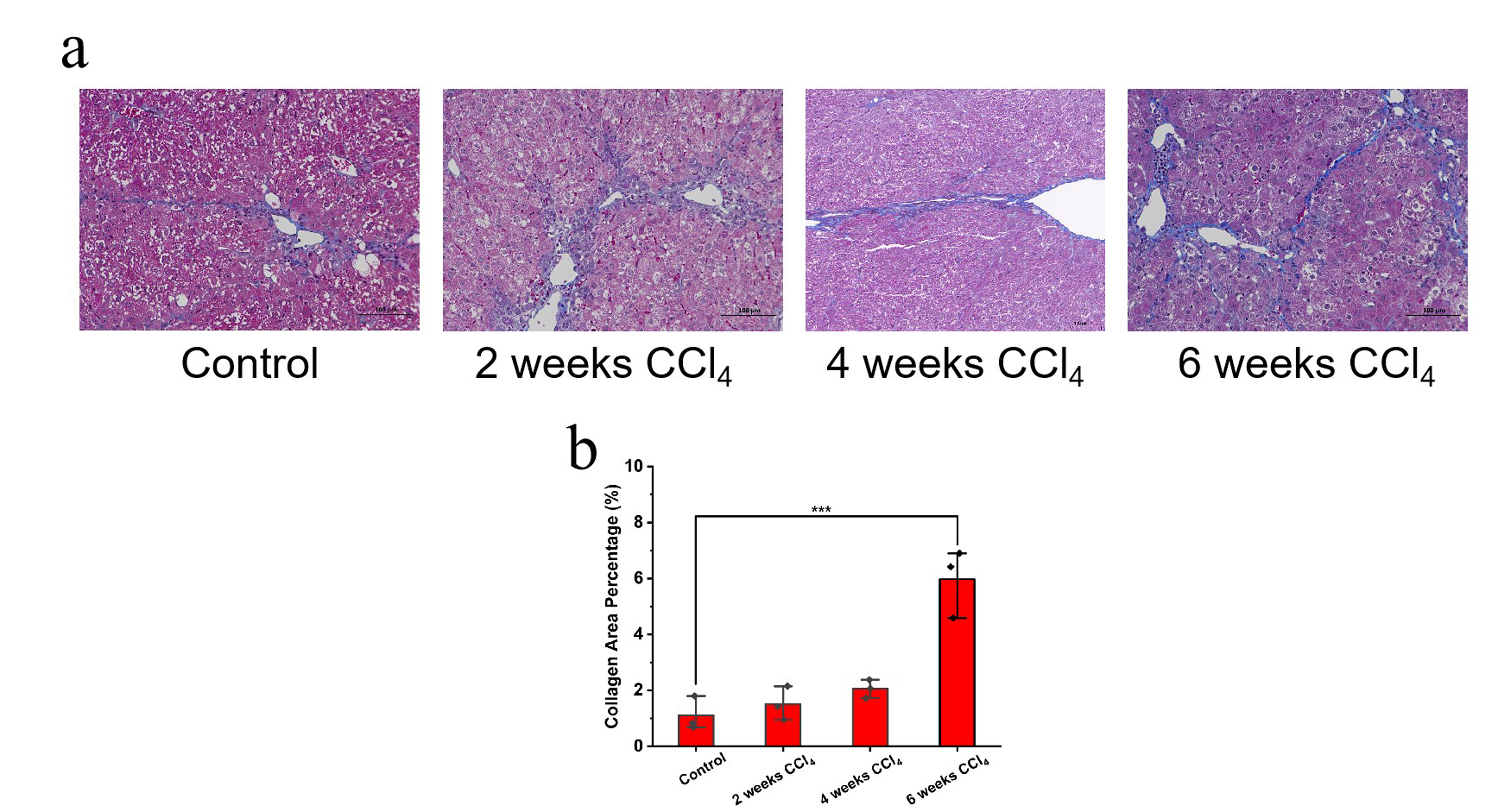


Supplementary Figure 16. The Masson trichrome stain experiment of the mice with various levels of aHSCs. Mice were intraperitoneally injected with CCl_4_ solution (1.0 mL kg^-1^) twice a week for 2 weeks, 4 weeks, and 6 weeks, respectively. The control mice were intraperitoneally injected with olive oil for 6 weeks. (a) The Masson trichromatic staining of HSCs with different activation levels in mice. (b) The proportion of collagen area of the Masson trichrome stain. Scale bar = 100 μm. The data were expressed as mean ± SD, n = 3. ***P < 0.001 compared to the control group.


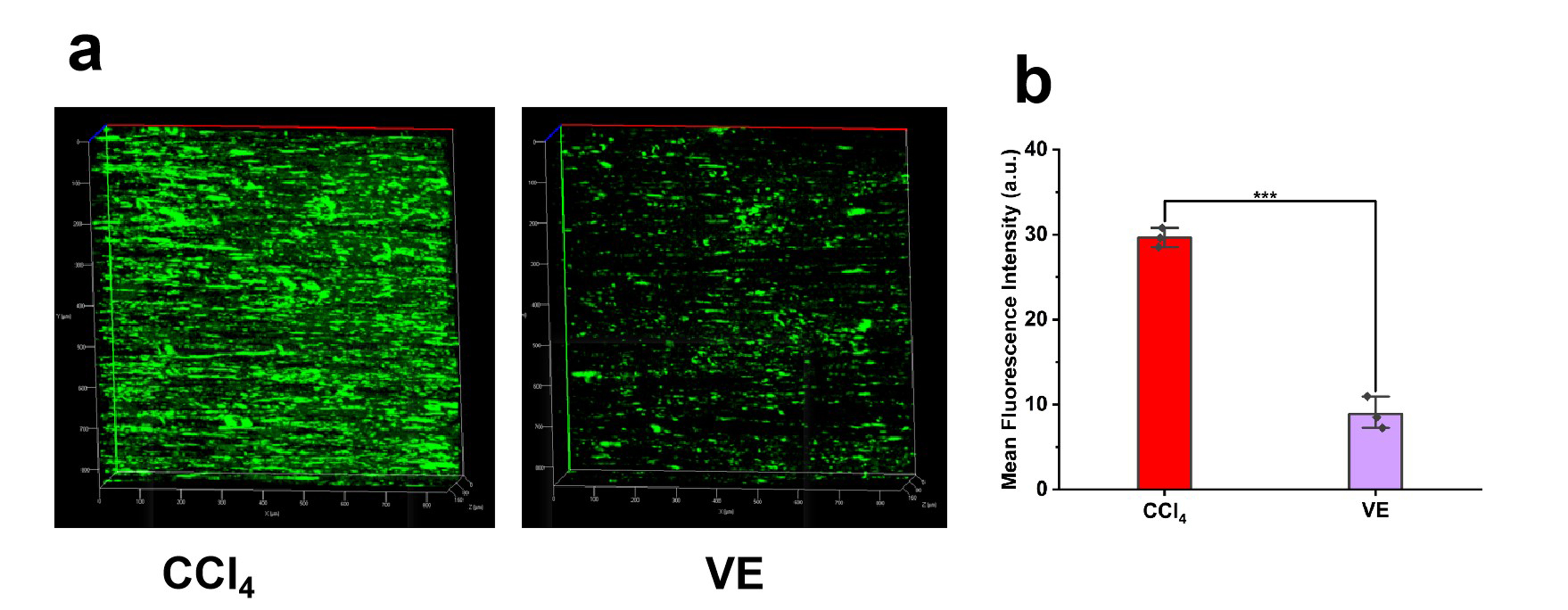


Supplementary Figure 17. Fluorescence imaging of mice with inhibition of HSCs activation. The mice were first given CCl_4_ solution for 6 weeks, followed by 2 weeks of either stroke-physiological saline solution (SPSS) or VE. (a) SPSS and VE. (b) Fluorescence intensities of a. The data were expressed as mean ± SD, n = 3. ***P < 0.001 compared to the control group.


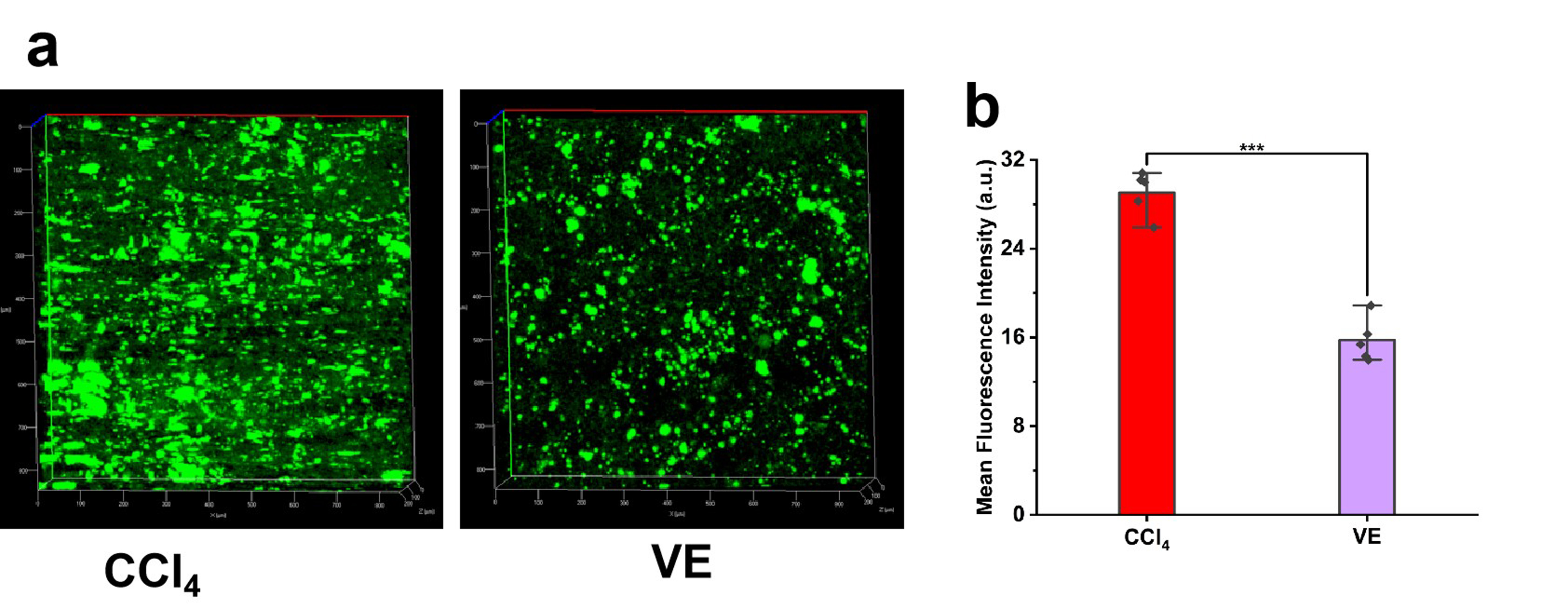


Supplementary Figure 18. Fluorescence imaging of mice with of HCC mice with the inhibited aHSCs by VE. (a) SPSS and VE. (b) Fluorescence intensities of a. The data were expressed as mean ± SD, n = 5. ***P < 0.001 compared to the control group.


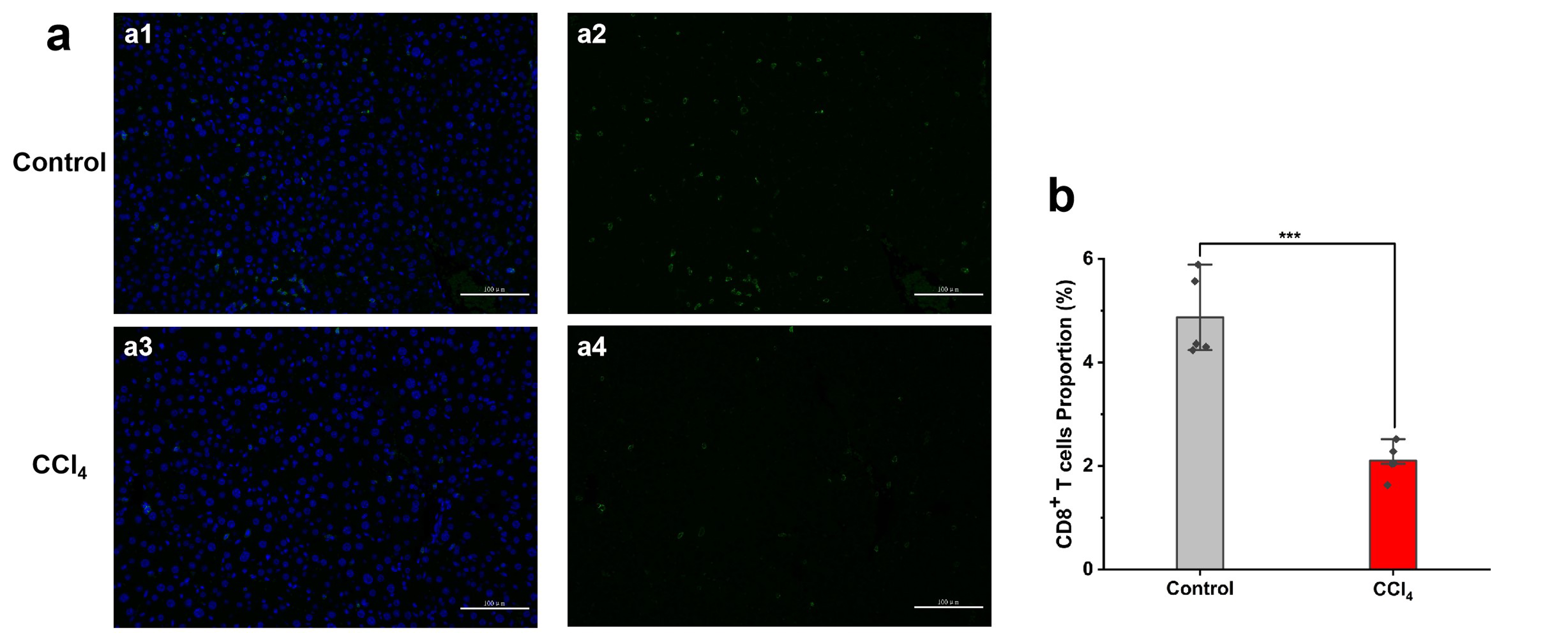


Supplementary Figure 19. The immunofluorescence staining experiments of the orthotopic mouse models of HCC containing q-HSCs or aHSCs. (a) The immunofluorescence staining results. Control group: the orthotopic mouse models of HCC containing q-HSCs. CCl_4_ group: the orthotopic mouse models of HCC containing aHSCs. Blue: hepatic cells. Green: CD8^+^ T cells. (b) The proportions of CD8^+^ T cells around the tumor. Scale bar = 100 μm. The data were expressed as mean ± SD, n = 5. ***P < 0.001 compared to the control group.


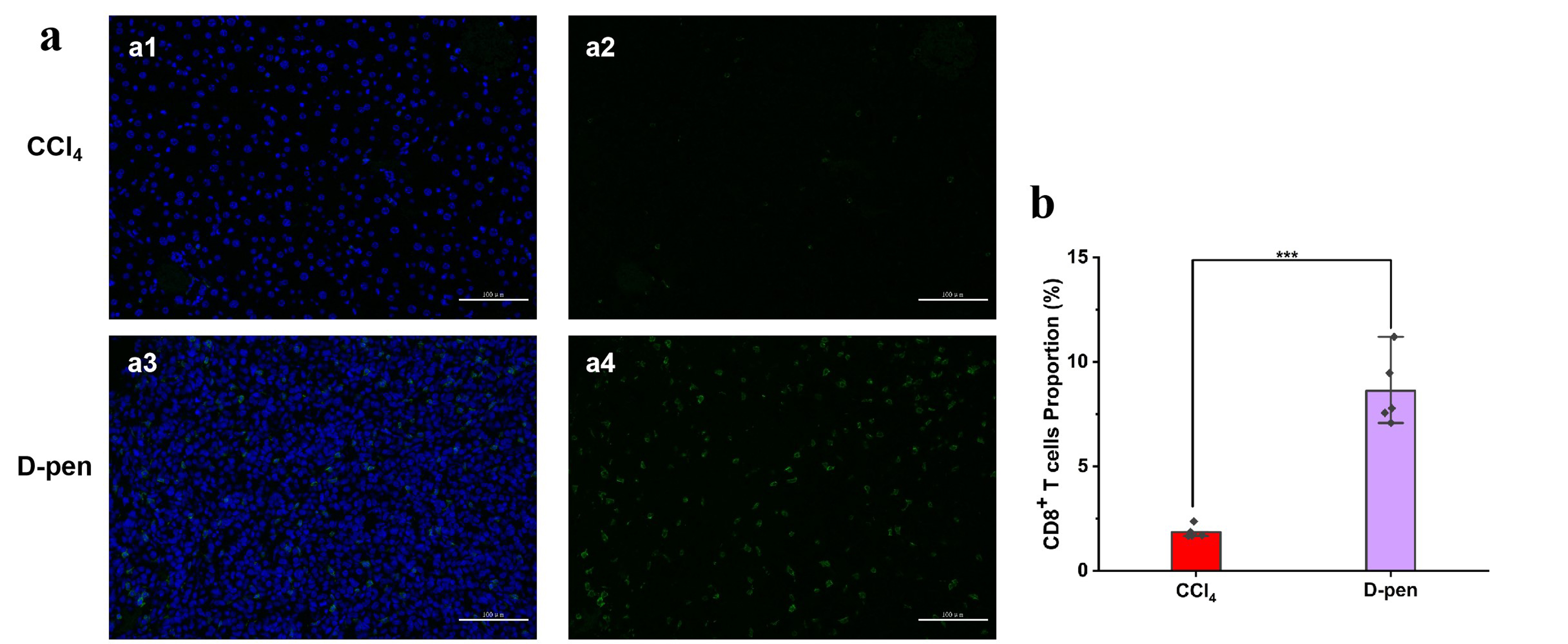


Supplementary Figure 20. The immunofluorescence staining experiments of the orthotopic mouse models of HCC with the inhibited aHSCs by D-pen. (A) The immunofluorescence staining results. Control group: the orthotopic mouse models of HCC with aHSCs. D-pen group: D-pen was used to inhibit aHSCs. Blue: hepatic cells. Green: CD8^+^ T cells. (B) The proportions of CD8^+^ T cells around the tumor. Scale bar = 100 μm. The data were expressed as mean ± SD, n = 5. ***P < 0.001 compared to the control group.


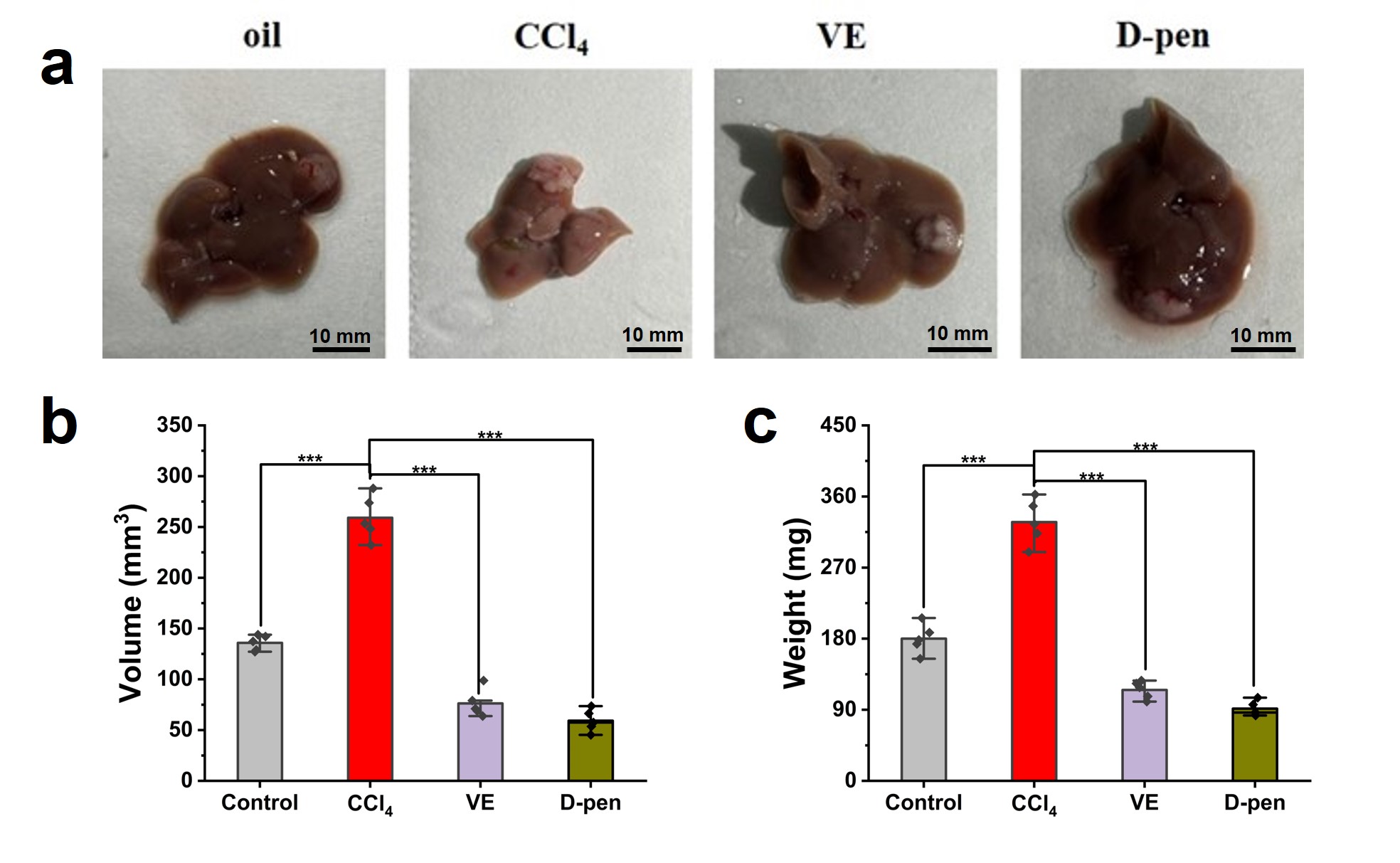


Supplementary Figure 21. Effect of the aHSCs on immune evasion to HCC. (A) HCC growth in mice with different activation levels of HSCs. Oil: control HCC mice. CCl_4_ (experimental group): CCl_4_ was injected twice a week for 4 weeks, and then the orthotopic mouse model was constructed. VE or D-pen (inhibitory group): after HCC mice with aHSCs-HCC, they were treated by injections of VE or D-pen, which were injected 3 times a week for 2 weeks. All mice were grown for 2 weeks after constructing in situ tumour, and tumour weight and volume were subsequently measured. B and C: tumour volume and weight in mice. Scale bar = 10 mm. The data were expressed as mean ± SD, n = 5. ***P < 0.001 compared to the CCl_4_ group.


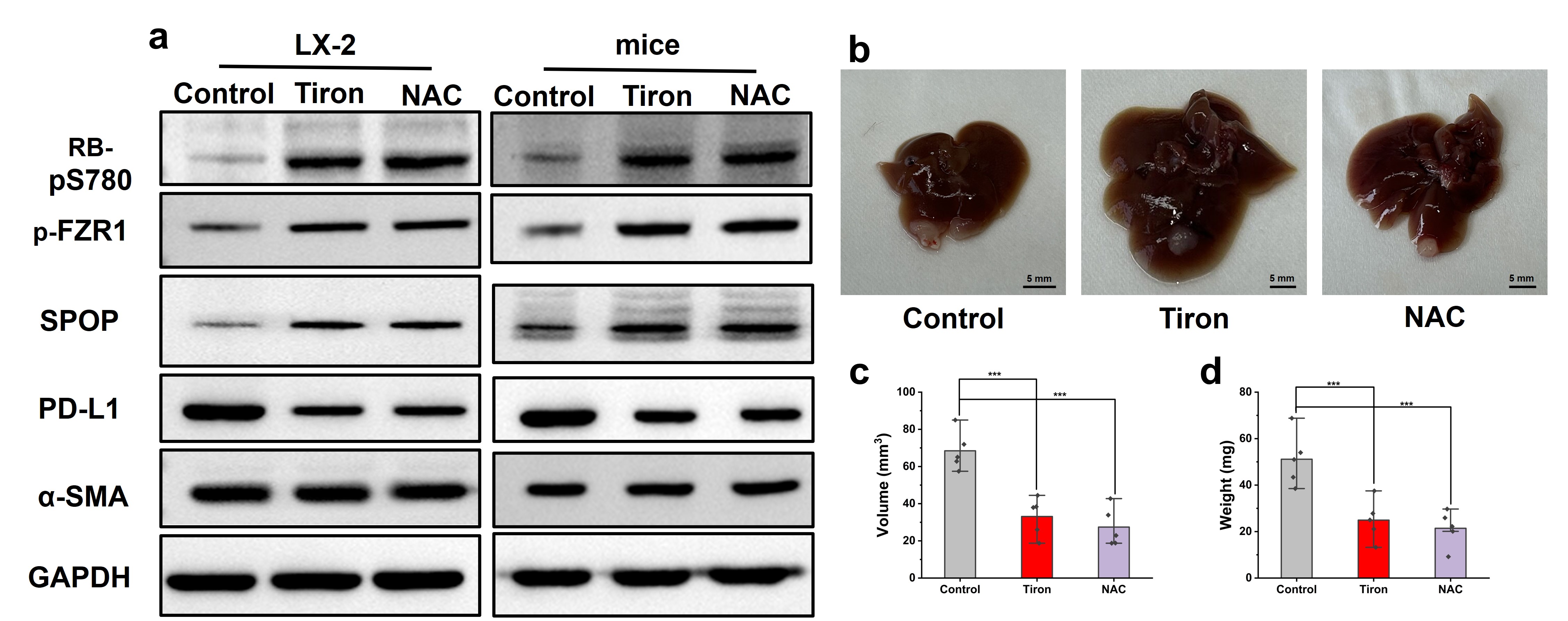


Supplementary Figure 22. ROS promotes immune evasion from HCC. a: Western blotting of LX-2 cells and mice with different ROS level. Control: TGF-β1 (5 ng mL^-1^) was incubated for 12 h. Tiron: TGF-β1 was incubated and Tiron (10 µM) was added for 12 h. NAC: TGF-β1 was incubated and NAC (10 µM) was added for 12 h. b: HCC growth in mice with different ROS levels. Control: do no processing. Tiron or VE: mice were injected intraperitoneally with Tiron or VE (1mM), which were injected 3 times a week. All mice were grown for 10 days after the construction of in situ tumour, followed by measurement of tumour weight and volume. c and d: tumour volume and weight in mice. Scale bar = 5 mm. The data were expressed as mean ± SD, n = 5. **p< 0.05, ***P < 0.001 compared to the control group.


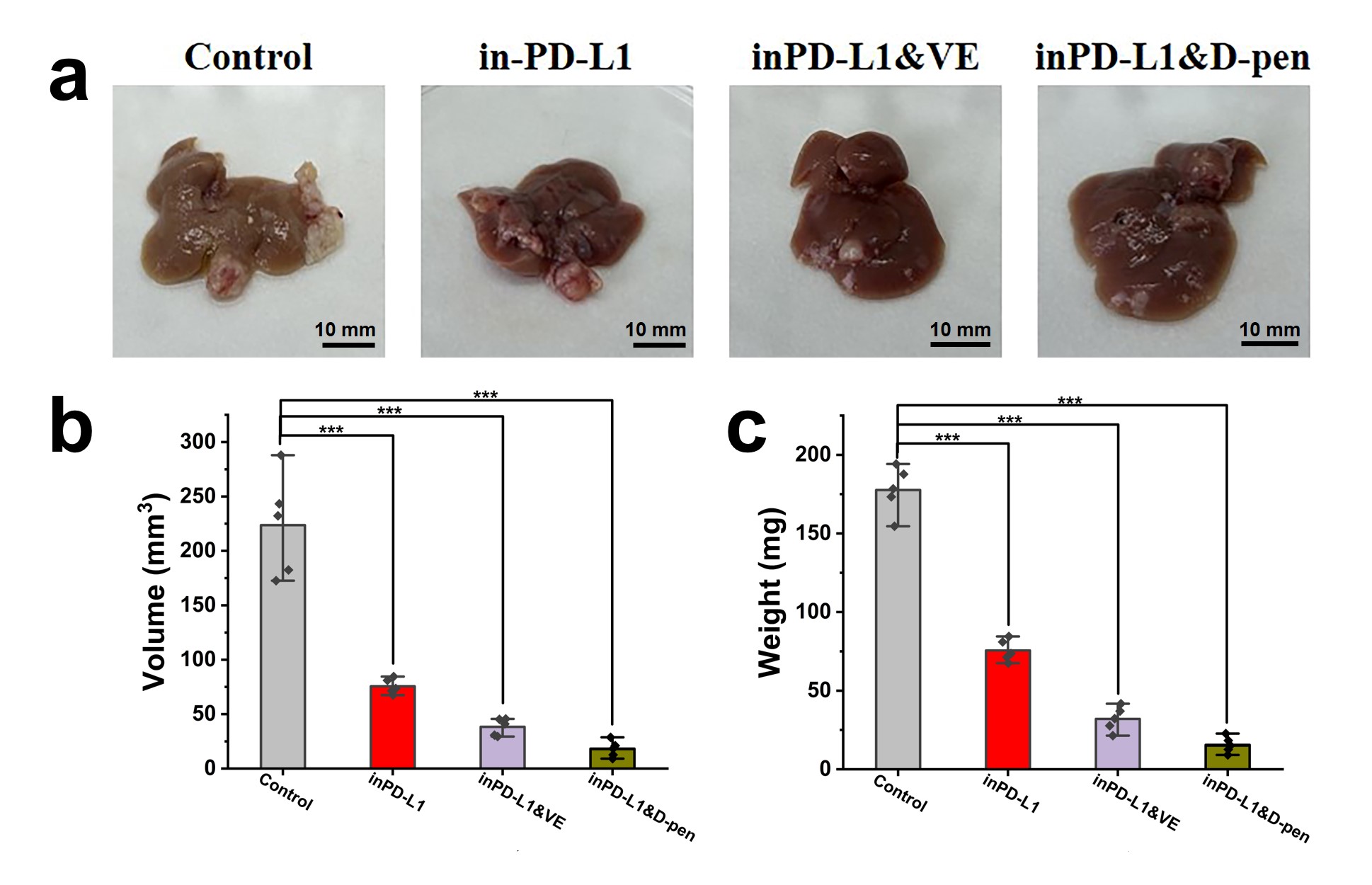


Supplementary Figure 23. PD-L1 blocking antibody treatment experiments. (a) Effects of different inhibitors on tumour growth. First, mouse models with aHSCs-HCC were constructed, followed by treatment with different inhibitors. Control: do no processing. Experimental group (inPD-L1): treated with PD-L1 inhibitor (BMS-1, HY-19991, MedChemexpress), 50 μg / 3 days for 3 times. Inhibition groups (inPD-L1&VE, inPD-L1&D-pen) were treated with a combination of PD-L1 blocker and VE (or D-pen).VE or D-pen was treated by intraperitoneal injections, 3 times / week for 2 weeks. All mice were grown for a fortnight after construction of in situ tumour, and tumour weights and volumes were subsequently measured. b and c: tumour volume and weight in mice. Scale bar = 10 mm. The data were expressed as mean ± SD, n = 5. ***P < 0.001 compared to the control group.


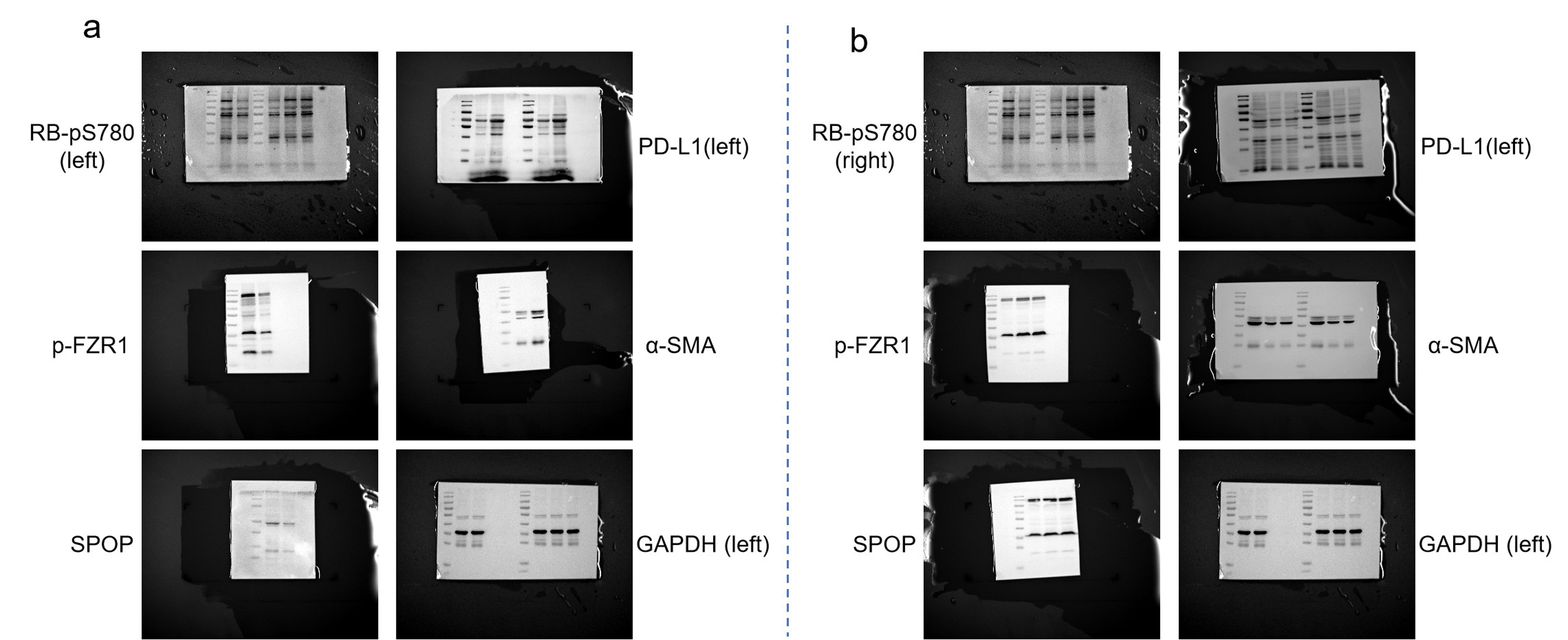


Supplementary Figure 24. Figure 7 uncropped and unedited blot images. a:Figure 7a. b: Figure 7b.
