# Supplementary material for: Fluorescence imaging sheds light on the immune evasion mechanisms of hepatic stellate cells mediated by superoxide anion
Source: Commun Biol. 2024 May 10;7:558. doi: 10.1038/s42003-024-06245-y (PMC11087649; doi:10.1038/s42003-024-06245-y)
Supplement: Supplementary file 2 — Description of Additional Supplementary Files [file 42003_2024_6245_MOESM2_ESM.docx]

Description of Additional Supplementary Files

File Name: Supplementary Data 1

Description: Source data underlying figures
