# Supplementary material for: Fluorescence imaging sheds light on the immune evasion mechanisms of hepatic stellate cells mediated by superoxide anion
Source: Commun Biol. 2024 May 10;7:558. doi: 10.1038/s42003-024-06245-y (PMC11087649; doi:10.1038/s42003-024-06245-y)
Supplement: Supplementary file 4 — Reporting Summary [file 42003_2024_6245_MOESM4_ESM.pdf]

nature portfolio

Corresponding author(s): Bo Tang, DAPR, COMMSBIO-22-4340B

Last updated by author(s): Feb 12, 2024

Reporting Summary

Nature Portfolio wishes to improve the reproducibility of the work that we publish. This form provides structure for consistency and transparency in reporting. For further information on Nature Portfolio policies, see our Editorial Policies and the Editorial Policy Checklist.

Statistics

For all statistical analyses, confirm that the following items are present in the figure legend, table legend, main text, or Methods section.

n/a	Confirmed	
☐	☒	The exact sample size (*n*) for each experimental group/condition, given as a discrete number and unit of measurement
☐	☒	A statement on whether measurements were taken from distinct samples or whether the same sample was measured repeatedly
☐	☒	The statistical test(s) used AND whether they are one- or two-sided<br>*Only common tests should be described solely by name; describe more complex techniques in the Methods section.*
☐	☒	A description of all covariates tested
☐	☒	A description of any assumptions or corrections, such as tests of normality and adjustment for multiple comparisons
☐	☒	A full description of the statistical parameters including central tendency (e.g. means) or other basic estimates (e.g. regression coefficient) AND variation (e.g. standard deviation) or associated estimates of uncertainty (e.g. confidence intervals)
☐	☒	For null hypothesis testing, the test statistic (e.g. *F*, *t*, *r*) with confidence intervals, effect sizes, degrees of freedom and *P* value noted<br>*Give P values as exact values whenever suitable.*
☒	☐	For Bayesian analysis, information on the choice of priors and Markov chain Monte Carlo settings
☒	☐	For hierarchical and complex designs, identification of the appropriate level for tests and full reporting of outcomes
☐	☒	Estimates of effect sizes (e.g. Cohen's *d*, Pearson's *r*), indicating how they were calculated

Our web collection on statistics for biologists contains articles on many of the points above.

Software and code

Policy information about availability of computer code

Data collection	The spectral characteristics of the probe were measured by F-4600 fluorescence spectrometer.<br>Fluorescence imaging results of cells and mice were obtained using LSM 880 NLO two-photon confocal microscopy.
Data analysis	Origin 2021 were used to analysed data.<br>Fluorescence imaging results of cells and mice were analyzed with ZEN 2.0 (blue edition).

For manuscripts utilizing custom algorithms or software that are central to the research but not yet described in published literature, software must be made available to editors and reviewers. We strongly encourage code deposition in a community repository (e.g. GitHub). See the Nature Portfolio guidelines for submitting code & software for further information.

Data

Policy information about availability of data

All manuscripts must include a data availability statement. This statement should provide the following information, where applicable:

- Accession codes, unique identifiers, or web links for publicly available datasets
- A description of any restrictions on data availability
- For clinical datasets or third party data, please ensure that the statement adheres to our policy

All data generated or analysed during this study are included in this published article (and its supplementary information files)

Research involving human participants, their data, or biological material

Policy information about studies with human participants or human data. See also policy information about sex, gender (identity/presentation), and sexual orientation and race, ethnicity and racism.

Reporting on sex and gender	No human study participants are involved in this manuscript.
Reporting on race, ethnicity, or other socially relevant groupings	No human study participants are involved in this manuscript.
Population characteristics	No human study participants are involved in this manuscript.
Recruitment	No human study participants are involved in this manuscript.
Ethics oversight	No human study participants are involved in this manuscript.

Note that full information on the approval of the study protocol must also be provided in the manuscript.

Field-specific reporting

Please select the one below that is the best fit for your research. If you are not sure, read the appropriate sections before making your selection.

☒ Life sciences ☐ Behavioural & social sciences ☐ Ecological, evolutionary & environmental sciences

For a reference copy of the document with all sections, see nature.com/documents/nr-reporting-summary-flat.pdf

Life sciences study design

All studies must disclose on these points even when the disclosure is negative.

Sample size	For all in vitro (cell) test, such as 3 samples are used to give average value with significant difference analysis. For all in vivo test, such as 5 samples are used to give average value with significant difference analysis;
Data exclusions	No data were excluded from the analyses
Replication	All in vitro result are reproducibility in the manuscript. For in vivo assay, 3-10 samples are used for different assays to give solid result with significant difference between control groups and test groups.
Randomization	All data were allocated randomly.
Blinding	Not applicable. All data were allocated randomly by the investigators

Reporting for specific materials, systems and methods

We require information from authors about some types of materials, experimental systems and methods used in many studies. Here, indicate whether each material, system or method listed is relevant to your study. If you are not sure if a list item applies to your research, read the appropriate section before selecting a response.

Materials & experimental systems

n/a	Involved in the study	
☐	☒	Antibodies
☐	☒	Eukaryotic cell lines
☒	☐	Palaeontology and archaeology
☐	☒	Animals and other organisms
☒	☐	Clinical data
☒	☐	Dual use research of concern
☒	☐	Plants

Methods

n/a	Involved in the study	
☒	☐	ChIP-seq
☒	☐	Flow cytometry
☒	☐	MRI-based neuroimaging

Antibodies

Antibodies used	Anti-PD-L1/CD274, BOSTER, PB0166, 1:1000; Anti-α-SMA, BOSTER, BM0002, 1:1000; Anti-SPOP, BOSTER, M02032, 1:1000; FZR1 Polyclonal Antibody, Thermo Fisher Scientific, 34-2000, 1:1000; Phospho-Rb (Ser780) Rabbit pAb, ZENBIO, 340792, 1:1000.

Validation	Anti-PD-L1/CD274, distributor website (https://www.boster.com.cn/index/products/productslist?fcatid=all&keywords=PB0166); Anti-α-SMA, distributor website (https://www.boster.com.cn/index/products/productslist?fcatid=all&keywords=BM0002); Anti-SPOP, distributor website (https://www.boster.com.cn/index/products/productslist?fcatid=all&keywords=M02032); FZR1 Polyclonal Antibody, distributor website (https://www.thermofisher.cn/cn/zh/antibody/product/FZR1-Antibody-Polyclonal/34-2000); Phospho-Rb (Ser780) Rabbit pAb, distributor website (http://www.zen-bio.cn/prod_view.aspx?IsActiveTarget=True&TypeId=180&Id=553880&FId=t3:180:3).

Eukaryotic cell lines

Policy information about cell lines and Sex and Gender in Research

Cell line source(s)	LX-2, 7702, HepG2, Hepa 1-6, RAW264.7, and C166 were purchased from Procell Life Science & Technology Co., Ltd. (Wuhan, China)
Authentication	Authentication of every cell line used in this study was conducted by short tandem repeat (STR) DNA Profiling. For each cell line used here, we always check its shape, growth condition and any contaminants by observing microscope. General cell maintenance is around 48 hr interval, suggesting normal cell proliferation.
Mycoplasma contamination	No mycoplasma contamination was detected according to report by supplier.
Commonly misidentified lines (See ICLAC register)	None. We confirmed that commonly misidentified lines in ICL AC have not been used in this study.

Animals and other research organisms

Policy information about studies involving animals; ARRIVE guidelines recommended for reporting animal research, and Sex and Gender in Research

Laboratory animals	C57BL/6 mouse, male, 4-6 weeks old
Wild animals	C57BL/6 mouse, male, 4-6 weeks old
Reporting on sex	We performed only male mice because of susceptibility to hepatocarcinogenesis.
Field-collected samples	The study did not involve samples collected from the field.
Ethics oversight	Animal Care Committee of Shandong Normal University

Note that full information on the approval of the study protocol must also be provided in the manuscript.

Plants

Seed stocks	No plant study are involved in this manuscript.
Novel plant genotypes	No plant study are involved in this manuscript.
Authentication	No plant study are involved in this manuscript.
